# Comparative epigenomics in the Brassicaceae reveals two evolutionarily conserved modes of PRC2-mediated gene regulation

**DOI:** 10.1186/s13059-017-1333-9

**Published:** 2017-10-31

**Authors:** Claudia Chica, Alexandra Louis, Hugues Roest Crollius, Vincent Colot, François Roudier

**Affiliations:** 1 Institut de Biologie de l’Ecole Normale Supérieure (IBENS), Ecole Normale Supérieure, Centre National de la Recherche Scientifique (CNRS), Institut National de la Santé et de la Recherche Médicale (INSERM), Paris, F-75005 France; 2 Present address: Institut Pasteur, Bioinformatics and Biostatistics Hub, C3BI, USR 3756 IP CNRS, Paris, France; 3 Present address: Laboratoire Reproduction et Développement des Plantes, Univ Lyon, ENS de Lyon, UCB Lyon 1, CNRS, INRA, F-69342, Lyon, France

**Keywords:** Comparative epigenomics, H3K27me3, Gene orthologs, PRC2, Chromatin, Chromosome architecture, Evolution, *Arabidopsis* sp, *Arabis alpina*, Brassicaceae

## Abstract

**Background:**

Polycomb Repressive Complexes 2 (PRC2) are multi-protein chromatin modifiers that are evolutionarily conserved among eukaryotes and play key roles in the regulation of gene expression, notably through the trimethylation of lysine 27 of histone H3 (H3K27me3). Although PRC2-mediated gene regulation has been studied in many organisms, few studies have explored in depth the evolutionary conservation of PRC2 targets.

**Results:**

Here, we compare the H3K27me3 epigenomic profiles for the two closely related species *Arabidopsis thaliana* and *Arabidopsis lyrata* and the more distant species *Arabis alpina*, three Brassicaceae that diverged from each other within the past 24 million years.

Using a robust set of gene orthologs present in the three species, we identify two classes of evolutionarily conserved PRC2 targets, which are characterized by either developmentally plastic or developmentally constrained H3K27me3 marking across species. Constrained H3K27me3 marking is associated with higher conservation of promoter sequence information content and higher nucleosome occupancy compared to plastic H3K27me3 marking. Moreover, gene orthologs with constrained H3K27me3 marking exhibit a higher degree of tissue specificity and tend to be involved in developmental functions, whereas gene orthologs with plastic H3K27me3 marking preferentially encode proteins associated with metabolism and stress responses. In addition, gene orthologs with constrained H3K27me3 marking are the predominant contributors to higher-order chromosome organization.

**Conclusions:**

Our findings indicate that developmentally plastic and constrained H3K27me3 marking define two evolutionarily conserved modes of PRC2-mediated gene regulation that are associated with distinct selective pressures operating at multiple scales, from DNA sequence to gene function and chromosome architecture.

**Electronic supplementary material:**

The online version of this article (doi:10.1186/s13059-017-1333-9) contains supplementary material, which is available to authorized users.

## Background

The first Polycomb Repressive Complex 2 (PRC2) was identified in *Drosophila melanogaster (Dm)*, where it is essential for maintaining the repressed state of homeotic genes [1, 2]. *Dm* PRC2 contains four core subunits, including the histone methyltransferase Enhancer of zeste, E(z), which catalyzes the trimethylation of lysine 27 of histone H3 (H3K27me3). PRC2 is present in most eukaryotes, including mammals and plants [1, 3]. In the flowering plant *Arabidopsis thaliana*, PRC2-mediated regulation of gene expression is involved in key developmental decisions throughout the life cycle, including postembryonic growth following seed germination and flowering [4].

In contrast to *D. melanogaster*, which contains a single E(z) and thus a single PRC2 holoenzyme, there are three E(z)-like proteins in *Arabidopsis thaliana*, called MEDEA (MEA), SWINGER (SWN), and CURLY LEAF (CLF). Whereas the gene encoding MEA is expressed exclusively in the endosperm, those encoding SWN and CLF have broad and overlapping expression patterns [4]. Together, SWN and CLF are likely responsible for all H3K27me3 marking outside of the endosperm, and this marking affects around 30% of *Arabidopsis thaliana* genes in total, based on epigenomic maps obtained for multiple developmental stages and tissues [5]. In agreement with the critical role of PRC2 in orchestrating plant development and growth, H3K27me3-marked genes are notably enriched in transcription factors (TFs) as well as in components of hormone signaling [6–9].

Although regulation of gene expression by PRC2 is an ancestral eukaryotic invention [1, 3], the degree to which H3K27me3 marking is conserved between gene orthologs has not been precisely determined, and the selective pressures underlying this conservation are not known.

The few comparative epigenomic studies that have been performed to date in plants and animals indicate that H3K27me3 marking tends to be concordant among gene orthologs [10–13]. In contrast, recent gene duplicates often exhibit discordant H3K27me3 marking [11, 12, 14]. Furthermore, work in *Arabidopsis thaliana* revealed that the few recent gene duplicates with concordant H3K27me3 marking are characterized by an enrichment in conserved non-coding sequences (CNSs) [15]. Taken together, these findings point on the one hand to a strong evolutionary conservation of PRC2 regulation and on the other hand to its differential use among gene duplicates, consistent with the latter being typically subjected to relaxed or diversifying selection.

To gain further insights into the evolutionary conservation of PRC2 targeting, we set out to compare H3K27me3 marking between gene orthologs in the three Brassicaceae species *Arabidopsis thaliana (Ath), Arabidopsis lyrata (Aly)*, and *Arabis alpina (Aal). Ath* and *Aly* diverged from each other approximately 6 million years ago (MYA), while the last common ancestor with *Aal* dates back to around 24 MYA [16, 17], thus enabling us to evaluate conservation over distinct time scales. Central to our study is the establishment, based on the reconstruction of the ancestral Brassicaceae genome, of a robust set of 13,515 gene orthologs that are present in the three species. By combining this gene set and chromatin immunoprecipitation sequencing (ChIP-seq) data obtained using matched samples for the three species, we uncovered the existence of two distinct modes of evolutionarily conserved H3K27me3 marking, which are either developmentally plastic or developmentally constrained across species. We demonstrate that constrained marking is characterized by higher conservation of sequence composition at promoters, higher nucleosome occupancy, and more pronounced tissue-specific expression compared to plastic marking. We also show that among gene orthologs marked by H3K27me3, those with constrained marking contribute more to the most frequent intra-chromosomal interactions. These and additional findings indicate that the H3K27me3 epigenomic landscape of individual Brassicaceae species, and presumably of other plants as well, is shaped by two main classes of evolutionarily conserved PRC2 targets that are subjected to distinct selective pressures operating at multiple scales, from DNA sequence to gene function and chromosome architecture.

## Results

### Defining robust sets of gene orthologs in the Brassicaceae

**Fig. 1:**
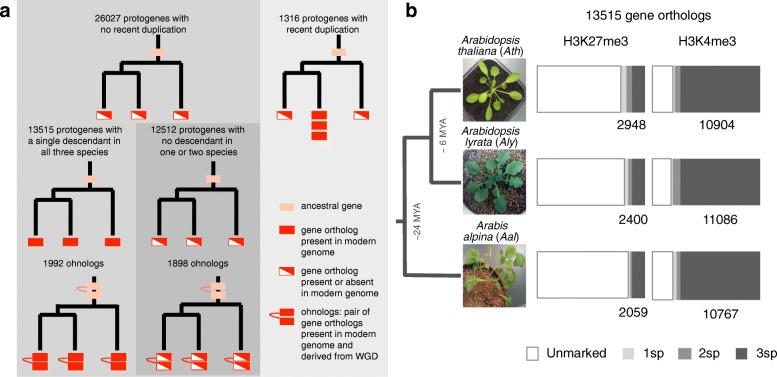
Gene orthologs and their chromatin marking across *Ath*, *Aly* and *Aal*. **a** Schematic representation of the different sets of gene orthologs defined based on 27,343 Brassicaceae protogenes. **b** H3K27me3 and H3K4me3 marking for the 13,515 gene orthologs that correspond to single gene descendants in all three species. The number of marked genes is indicated for each species

In order to assess rigorously the conservation of H3K27me3 marking between gene orthologs, we first established a robust comparative framework. Initial work based on the genome sequence of *Ath*, *Aly*, *Capsella rubella*, *Brassica rapa*, and *Thellungiella parvula* led to the reconstruction of the ancestral Brassicaceae karyotype with 30,968 protogenes [16]. Since then, the genome of two additional Brassicaceae has been sequenced (*Aal* and *Tarenaya hassleriana* [18, 19]). Using this extended dataset as well as the genome sequence of two outgroup species, we now define 27,343 Brassicaceae protogenes with descendants in *Ath*, *Aly*, or *Aal* (see Methods; Fig. 1a). This set comprises 26,027 protogenes for which the descendants were not subjected to recent duplication in any of the three species. Specifically, there are 13,515 protogenes with a single gene descendant in all three species and 12,512 protogenes with no descendant in one or two species (Fig. 1a). The remaining 1316 protogenes are distinguished from the two main subgroups by the presence of recent duplicates (i.e., recent paralogs) in at least one of the three species. The descendants of the first subgroup of 13,515 protogenes served to analyze the concordance of chromatin marking between the three species, while the descendants of the other two subgroups served to investigate the relationship between H3K27me3 marking and recent gene duplication or loss.

### H3K27me3 marking of gene orthologs tends to be concordant between *Ath*, *Aly*, and *Aal*

We previously generated epigenomic maps of H3K27me3 and H3K4me3 (a mark typically associated with transcriptionally active genes) as well as transcriptome profiles from matched leaf samples of *Ath*, *Aly*, and *Aal* [18]. We showed that genes marked by H3K27me3 have lower expression on average than genes marked by H3K4me3 in all three species, as expected, and that these have similar epigenomic landscapes [18]. Specifically, 24%, 26%, and 30% of all annotated genes are marked by H3K27me3, 63%, 65%, and 70% are marked by H3K4me3 instead, and few genes exhibit both marks in *Ath*, *Aly* and *Aal*, respectively (Additional file 1: Table S1). Moreover, H3K27me3 is usually distributed broadly over individual transcription units, while H3K4me3 is preferentially located over the 5’ end of genes in all three species (Additional file 1: Figure S1).

To assess the level of conservation of chromatin marking among gene orthologs in *Ath*, *Aly*, and *Aal*, we focused on those that correspond to the set of 13,515 protogenes with a single descendant in all three species. Overall, 3673 (27%) and 11,764 (87%) of these gene orthologs are marked in at least one species by H3K27me3 and H3K4me3, respectively (Table 1). Remarkably, while almost all (93%) H3K4me3-marked gene orthologs show concordant marking (marking in at least two of the three species), this percentage drops to 62% for H3K27me3-marked gene orthologs (Fig. 1b, Table 1). Moreover, among the 3673 gene orthologs marked by H3K27me3, those marked in only one species or in all three species are much more abundant than expected by chance (38% vs 22% for single-species marking and 40% vs 30% for three-species marking; Additional file 1: Table S2). Likewise, out of the 806 gene orthologs marked in only two species, a disproportionate number (512, 64%) are marked in the two closely related species *Ath* and *Aly* (Table 1). These results indicate therefore that discordant H3K27me3 marking does not primarily reflect technical limitations in our ability to detect this chromatin mark equally well across the three species and point instead to the existence of two distinct classes of gene orthologs marked by H3K27me3. Consistent with this notion, gene orthologs with concordant H3K27me3 marking have a much higher proportion of their maize and rice counterparts that exhibit this mark [11] than gene orthologs with discordant marking (26% vs 15%, Table 1).

**Table 1:** Concordance of chromatin marking across species

Species with marking	H3K4me3	H3K27me3	H3K27me3 also in maize and rice
*Ath-Aly-Aal*	10,010	1464	384
*Ath-Aly*	505	512	68
*Aly-Aal*	280	97	10
*Ath-Aal*	188	197	31
*Ath*	201	775	121
*Aly*	291	327	44
*Aal*	289	301	46
Unmarked	1751	9842	NA
Total	13,515	13,515	NA

Numbers refer to gene orthologs

We therefore partitioned the 13,515 gene orthologs into two classes. Specifically, gene orthologs that in our matched leaf samples are marked by H3K27me3 in one or the two *Arabidopsis* species only or exclusively in *Aal* define a first class of PRC2 targets, with lineage-specific or “plastic” marking. Conversely, gene orthologs that in our matched leaf samples are marked in *Aal* and in at least one of the *Arabidopsis* species define the class of PRC2 targets with “constrained” marking, which in a parsimonious scenario corresponds to a developmentally invariant marking since the Brassicaceae ancestor. Further support for this classification comes from the fact that gene orthologs with plastic and constrained H3K27me3 marking are preferentially distributed in separated gene trees among the 10,480 ancestral Brassicaceae gene families (see Methods; Additional file 1: Table S3).

### Constrained H3K27me3 marking is associated with conservation of sequence information specifically within promoters

**Fig. 2:**
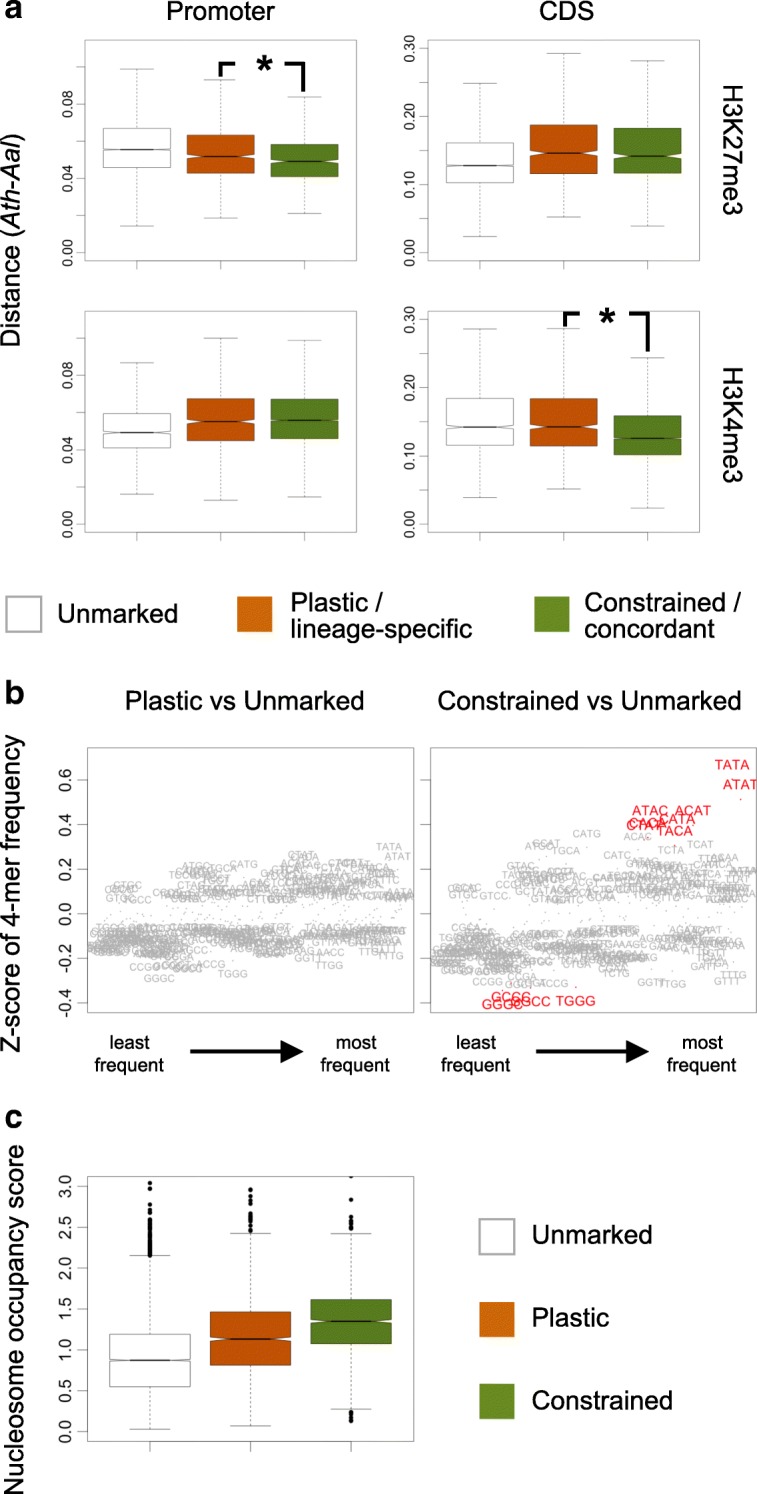
Sequence conservation and nucleosome occupancy of gene orthologs according to their chromatin marking status across species. **a** Promoter and coding sequence similarity (*Ath*-*Aal* comparisons) between gene orthologs according to their H3K27me3 or H3K4me3 marking status across species (plastic or constrained for H3K27me3 and lineage-specific or concordant for H3K4me3 marking). Only the 13,515 gene orthologs that correspond to single gene descendants in all three species are considered. Asterisks indicate significant variations of sequence similarity in relation to chromatin marking status across species (analysis of variance (ANOVA), *p* value < 10e-10). **b** Sequence enrichment (4-mers) for the promoters of the 1915 and 1758 gene orthologs with plastic or constrained H3K27me3 marking in comparison to the 9842 unmarked gene orthologs. Positive and negative Z-score values indicate higher and lower frequencies of 4-mers in comparison to unmarked gene orthologs (4-mers showing highest/lowest frequency are highlighted in *red*). **c** Nucleosome occupancy score for the 13,515 gene orthologs according to their H3K27me3 marking status

To determine if gene orthologs with plastic and constrained H3K27me3 marking differ in their degree of sequence conservation, we measured the sequence distance (see Methods) in the three pairwise comparisons, *Ath-Aly*, *Ath-Aal*, and *Aly-Aal*, for the promoter region (500 bp upstream of the transcription start site) as well as for the exons of marked gene orthologs. This analysis revealed that constrained H3K27me3 marking is associated with a lower sequence distance specifically over the promoter region (Fig. 2a, Additional file 1: Table S4). Conversely, gene orthologs with concordant rather than lineage-specific H3K4me3 marking show a higher degree of conservation specifically over the coding sequence (Fig. 2a, Additional file 1: Table S4). Moreover, gene orthologs with concordant H3K4me3 marking are the most active transcriptionally (Additional file 1: Figure S2), in keeping with the positive correlation broadly observed between protein sequence conservation and expression level [20].

To investigate further the differential degree of promoter sequence similarity between gene orthologs with plastic or constrained H3K27me3 marking, we determined the frequency of 4-mer sequences for these two groups (see Methods). This analysis was carried out independently for each of the three species to account for possible differences in genome sequence composition. Importantly, whether associated with plastic or constrained H3K27me3 marking, promoters have comparable information content as measured by Shannon entropy (Additional file 1: Figure S3). Nonetheless, 4-mer frequencies differ significantly between promoters of gene orthologs with plastic or constrained H3K27me3 marking. Thus, promoters of the latter group have respectively higher and lower proportions of AT/TA/AC/CA-rich and CC/GG/CG-rich 4-mers compared to promoters of gene orthologs unmarked in all three species. In contrast, promoters of gene orthologs with plastic H3K27me3 marking have 4-mer frequencies that are similar to those of gene orthologs with no H3K27me3 marking in any of the three species (Fig. 2b and Additional file 1: Figure S4 for *Aly* and *Aal*). Furthermore, the AT richness observed in the three species for the promoters of genes with constrained H3K27me3 marking does not result from a higher density of transposable elements (TEs), which are AT rich overall [21] (Additional file 1: Figure S5). Instead, these AT-richpromoters tend to have a higher nucleosome occupancy than those of gene orthologs that are either unmarked or with plastic H3K27me3 marking (Fig. 2c) in agreement with the observation that nucleosome occupancy positively correlates with A/T content in both *Ath* and rice [22].

Given their overall sequence conservation, we then searched for overrepresented sequence motifs in the promoters of all H3K27me3-marked gene orthologs. This analysis, which was carried out independently for the three species to account for possible differences in genome sequence composition, did not identify any sequence motif specifically enriched in the promoters of gene orthologs with constrained H3K27me3 marking. In contrast, GAGA and GAAGAA motifs, which were previously proposed to participate in PRC2 recruitment in *Ath* [23, 24], are overrepresented in the promoters of the two classes of H3K27me3-marked gene orthologs (Additional file 1: Table S5). This result suggests that gene orthologs with plastic H3K27me3 marking are as likely as those with constrained H3K27me3 marking to be *bona fide* PRC2 targets in all three species. In other words, rather than indicating a complete absence of PRC2 targeting in some species, plastic H3K27me3 marking would instead reflect developmental differences between species in the regulation of evolutionarily conserved PRC2 targets. To obtain direct evidence that this is the case, we took advantage of the H3K27me3 epigenomic maps that are available for additional tissues and organs in *Ath* (see Methods) and focused our analysis on the 725 gene orthologs that are unmarked in *Ath* but marked in either *Aly* or *Aal* in our matched leaf samples. Among these gene orthologs, 33% have H3K27me3 in at least one other tissue/organ in *Ath*, a much higher proportion than the one measured for the 9842 gene orthologs that are unmarked in all three species (11%) and for the 10,617 gene orthologs that are unmarked in *Aly* and *Aal* (8%). Given that this analysis is far from being exhaustive, we can confidently conclude that most gene orthologs with plastic H3K27me3 marking, like those with constrained H3K27me3 marking, are evolutionarily conserved PRC2 targets.

### Plastic and constrained H3K27me3 marking are associated with distinct gene functions and expression profiles

**Fig. 3:**
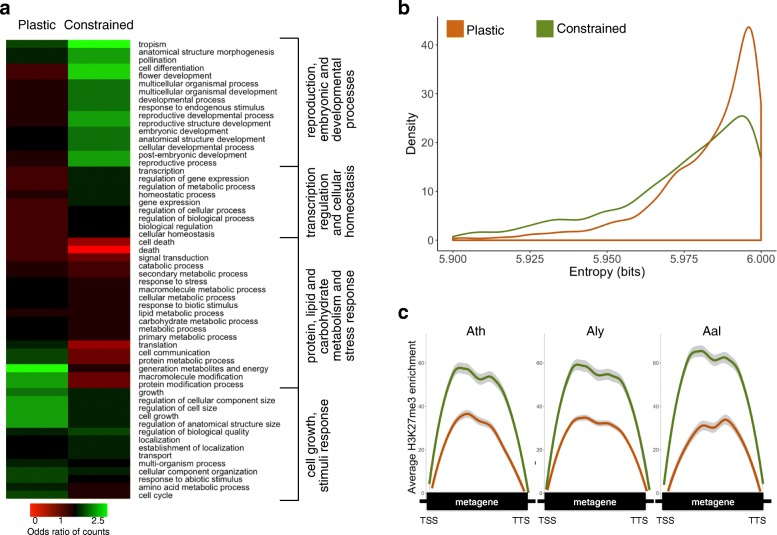
Functions, expression profiles, and H3K27me3 enrichment levels of gene orthologs with either plastic or constrained H3K27me3 marking. **a** Heatmap representation of normalized gene ontology (GO) term counts for the 1915 and 1758 gene orthologs with plastic or constrained H3K27me3 marking. *Red* and *green* indicate depletion and enrichment in comparison to all genes marked by H3K27me3, respectively. **b** Density plot of tissue-specific expression for gene orthologs with plastic or constrained H3K27me3 marking as estimated by Shannon entropy. The distributions are significantly different (Kolmogorov-Smirnov test *p* value < 0.05). Low entropy values indicate high tissue-specific expression. **c** Average H3K27me3 enrichment level over gene orthologs with plastic or constrained H3K27me3 marking. Color code is as in **b**

We next investigated the functions and expression patterns of gene orthologs with plastic or constrained H3K27me3 marking. Analysis of gene ontology revealed a clear distinction between these two classes, with the latter being enriched in categories related to transcription regulation, reproduction, and development. In contrast, gene orthologs marked in a developmentally plastic manner between species are enriched in categories related to basic metabolic processes such as carbohydrate and lipid synthesis, energy production, as well as cell growth and stress response (Fig. 3a). The two classes of gene orthologs are also clearly distinguished by their expression patterns in *Ath*, with developmentally constrained H3K27me3 marking being associated with a higher degree of tissue specificity (Fig. 3b). Moreover, average H3K27me3 enrichment is much higher over gene orthologs that show constrained H3K27me3 marking, and this is true for all three species (Fig. 3c).

**Fig. 4:**
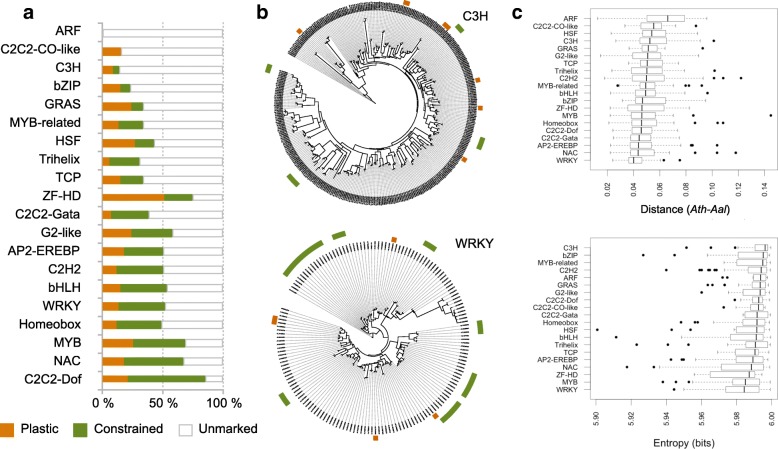
Plastic and constrained H3K27me3 marking among families of gene orthologs encoding transcription factors. **a** For each of the 20 largest gene families encoding transcription factors (TFs), the percentage of gene orthologs either not marked by H3K27me3 or else showing plastic or constrained H3K27me3 marking. **b** Phylogenetic trees showing the distribution of plastic or constrained H3K27me3 marking for the C3H and WRKY TF gene families. Color code is as in **a**. **c** Promoter sequence similarity (*Ath*-*Aal* comparisons, *top panel*) and tissue specificity of expression, as estimated by Shannon entropy for the 20 largest TF gene families (*bottom panel*)

As genes encoding TFs are noticeably enriched among PRC2 targets in plants [5], we analyzed further this functional category in relation to plastic or constrained H3K27me3 marking (see Methods). To allow meaningful comparisons, we considered only the 20 TF gene families with more than 10 members and for which at least 40% of them are among the 13,515 unambiguous gene orthologs defined initially. Nineteen of these TF gene families have at least one gene ortholog marked by H3K27me3 in *Ath* (Fig. 4a). Moreover, 67% of these gene orthologs (252 of 377 genes in total) show constrained H3K27me3 marking across the three species, a proportion that is almost twice that for the remaining 2571 non-TF gene orthologs that have H3K27me3 marking in *Ath* (37%). Nonetheless, large disparities exist among the 19 TF gene families: for instance, around 75% of marked gene orthologs belonging to the WRKY and C2C2-Dof families show constrained H3K27me3 marking, a proportion that drops to 0% and 46% for the marked gene orthologs that belong to the C2C2-CO-like and C3H families, respectively (Fig. 4a and b). Furthermore, and in agreement with the general trend described above, TF families that have the highest proportion of gene orthologs with constrained H3K27me3 marking (C2C2-Dof, NAC, MYB, Homeobox, and WRKY) show the highest level of promoter sequence similarity and tissue-specific expression (Fig. 4c).

### Gene orthologs with constrained H3K27me3 marking are important contributors to long-range intra-chromosomal interactions

Given the implication of H3K27me3 domains in the three-dimensional (3D) conformation of chromosomes in many eukaryotes including plants [25–28], we next investigated whether PRC2 targets with plastic and constrained marking could be distinguished by their chromosomal position or their implication in the spatial organization of the genome. We first analyzed the distribution of H3K27me3-marked genes along chromosomes in order to determine if constrained H3K27me3 marking could be related to a specific preservation of genomic location during the evolution of the Brassicaceae. To this end, we first identified blocks of synteny by comparing each modern genome to the ancestral Brassicaceae genome using PhylDiag [29]. In keeping with the high degree of conservation of genome organization among the Brassicaceae [16, 30], a large fraction of the descendants of the 27,343 protogenes could be assigned unambiguously to synteny blocks in *Aal, Aly*, and *Ath* (49%, 59%, and 63%, respectively), and most of the 13,515 gene orthologs are present in such blocks, irrespective of their marking. Thus, the two modes of H3K27me3 marking are not differentially associated with conservation of gene order among the Brassicaceae (Additional file 1: Table S6). However, the relatively short evolutionary distances that separate *Ath*, *Aly* and *Aal* might not be sufficient to detect differential association.

**Fig. 5:**
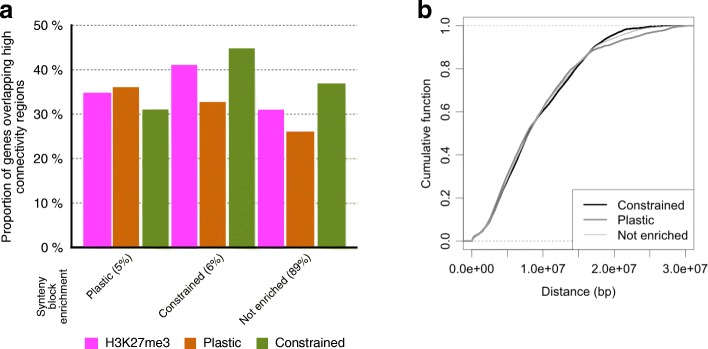
H3K27me3 marking of gene orthologs in relation to intra-chromosomal interactions in *Ath*. **a** Proportion of H3K27me3-marked genes overlapping with high connectivity regions as defined in Wang et al. [27] for synteny blocks enriched in genes with plastic or constrained H3K27me3 marking, or with no detectable enrichment. For each category of synteny blocks, the proportion of overlap is calculated separately for all the genes marked by H3K27me3 in *Ath* as well as for the genes with plastic or constrained H3K27me3 marking (see Methods). **b** Cumulative function distribution of the length of strong intra-chromosomal contacts involving synteny blocks either not enriched or else enriched in gene orthologs with either plastic or constrained H3K27me3 marking in the wild type

We next asked if plastic and constrained H3K27me3 marking contribute differently to these interactions. Using published high-resolution Hi-C data [27] and considering first all H3K27me3-marked gene orthologs, we found that they are generally enriched within high connectivity regions (32% of observed co-occurrence vs 22% expected at random). However, gene orthologs with constrained H3K27me3 marking co-occur significantly more frequently with high connectivity regions than those with plastic H3K27me3 marking (38% vs 32% for constrained gene orthologs and 27% vs 32% for plastic gene orthologs; Fisher exact test, *p* value < 0.05; Additional file 1: Table S8). The local density of H3K27me3-marked gene orthologs appears to be an important factor as well, since the co-occurrence of H3K27me3 marking with high connectivity regions is significantly higher for the synteny blocks that are enriched in gene orthologs with either plastic or constrained marking (36% vs 27% for the plastic category and 45% vs 38% for the constrained category; Fisher exact test, *p* value < 0.05; Fig. 5a; Additional file 1: Tables S7 and S8; Figure S6).

To explore further the importance of H3K27me3 marking in relation to the 3D organization of the genome, we analyzed another Hi-C dataset that provides information on the strength and distance of chromosomal interactions, both in wild-type (WT) and H3K27me3-depleted *clf swn* double mutant plants [26]. Using a broad classification (see Methods), interactions were ranked as weak, moderate, or strong (Additional file 1: Table S9). When comparing the distribution of contact distance in the WT for synteny blocks enriched in gene orthologs with plastic or constrained H3K27me3 marking, we found that the latter tend to be associated with a higher number of strong contacts in the 18–23 Mbp distance range (Fig. 5b). This distance range is similar to the size of *Ath* chromosomes, which therefore implies a specific contribution of orthologs with constrained H3K27me3 marking to strong intra-chromosomal interactions involving one or both ends of chromosomes. This distinction between the two classes of marked gene orthologs is barely detectable in the *clf swn* double mutant, which in addition shows a redistribution of strong intra-chromosomal contacts towards shorter distances (Additional file 1: Figure S7). However, because the *clf-28 swn-7* mutant used to generate the 2D interaction maps contains several chromosomal rearrangements [26], we cannot rule out that they contribute in part to these differences.

### Recent gene duplications are associated with *de novo* H3K27me3 marking

To assess comprehensively the association between PRC2-mediated regulation and the distinct evolutionary trajectories of genes, we next investigated the relationship between H3K27me3 marking and gene duplication or loss, two pervasive sources of genome evolution.

Gene duplicates present in extant Brassicaceae genomes have distinct evolutionary origins, some resulting from recent, mostly tandem duplications, and others being the remnants of the two ancestral whole genome duplications (WGDs) that took place 80 to 90 MYA (the β-WGD), before the emergence of the Brassicaceae, and approximately 40 MYA (the α-WGD), at the root of the Brassicaceae lineage [31, 32]. Thus, for each species, we compared the frequency of H3K27me3 marking between the 13,515 gene orthologs analyzed so far, the subset of 1992 gene orthologs for which we could establish that they are remnants of the α- or β-WGDs (the ohnologs, see Fig. 1 and Methods), and the additional group of 1316 gene orthologs that have been recently duplicated (i.e., duplicated after the Brassicaceae split in at least one of the three species considered; see Fig. 1). Recent duplicates show the highest frequency of H3K27me3 marking (36–47% vs 16–23% for the group of 13,515 gene orthologs, depending on the species; Additional file 1: Figure S8A). Moreover, H3K27me3 marking tends in this case to be restricted to the species that contains the gene duplicates (83% vs 48%; Additional file 1: Figure S8B; Table S10). These findings suggest that recent gene duplicates derive predominantly from ancestral genes that were not regulated by PRC2, and that tandem gene duplications are prone to acquire this type of regulation. In contrast, ohnologs are indistinguishable in terms of marking within and between species from the whole set of 13,515 gene orthologs (Additional file 1: Figure S8B; Table S10). Furthermore, pairs of ohnologs have a higher frequency than expected by chance to belong to the same class, i.e., to exhibit either plastic (4% vs 2%) or constrained H3K27me3 marking (8% vs 2%; chi-squared-test, *p* value < 0.0005), suggesting that WGDs do not lead to major alterations in PRC2 regulation.

Finally, we tested the association between H3K27me3 marking and recent gene loss. To this end, we compared the set of 12,512 gene orthologs with a missing counterpart in one or two species (see Fig. 1) to the set of 13,515 gene orthologs present in all three species. Results indicate that the proportion of gene orthologs marked by H3K27me3 is higher for the first set (24–38% vs 16–23%, depending on the species; Additional file 1: Figure S9A). Moreover, when gene loss affects one species only, marking is predominantly concordant in the two species that have retained the gene (Additional file 1: Figure S9B; Table S10). This high level of concordance cannot be explained by chance, since the subset of gene orthologs for which ohnologs can be identified show an opposite pattern, namely a prevalence of discordant marking between the two species that have retained the pair (Additional file 1: Figure S9B; Table S10). These findings therefore suggest that gene loss affects prevalently gene orthologs and ohnologs with constrained and plastic H3K27me3 marking, respectively.

## Discussion

Using comparative epigenomics and a refined reconstruction of the ancestral Brassicaceae genome that enabled us to derive robust sets of gene orthologs with distinct evolutionary trajectories, we have uncovered two broad classes of PRC2 targets. These two classes reflect invariant and lineage-specific use of gene regulation by PRC2 and correspond to two evolutionarily conserved modes of PRC2-mediated gene regulation. In other words, most gene orthologs with H3K27me3 marking in at least one Brassicaceae species likely correspond to ancestral PRC2 targets, which have been maintained as such during the evolutionary diversification of this plant family.

This last conclusion is supported by several lines of evidence. First, the majority of gene orthologs marked by H3K27me3 only in *Aly* and/or *Aal* are also targeted by PRC2 in *Ath* when considering tissues or organs other than the fully expanded leaves used for our comparative analysis, suggesting that the corresponding ancestral genes were already *bona fide* PRC2 targets. Second, gene orthologs with developmentally plastic and constrained H3K27me3 marking are preferentially distributed in separate gene trees among ancestral Brassicaceae gene families. Third, irrespective of the mode of H3K27me3 marking, gene orthologs have promoters that tend to be enriched in two motifs (GAGA and GAAGAA) that are proposed to participate in the recruitment of Polycomb Complexes in *Ath* [23, 24], which in this respect resemble the GAGA motif of animals [33, 34]. Fourth, H3K27me3 marking extends more often to maize and rice when this marking is constrained rather than plastic among the three Brassicaceae species that were analyzed.

The high degree of conservation of PRC2 targets between the Brassicaceae is in line with that documented within the *Drosophila* clade, which emerged at a similar time (~35 MYA) [12, 13]. It has been suggested that the typically broad patterns of H3K27me3 enrichment seen in *Drosophila* species, which define domains of up to ~100 kb covering several genes, may favor the conservation of PRC2 targets, despite sequence divergence [12, 13]. In contrast, H3K27me3 usually covers single genes in the Brassicaceae as well as in the other plants examined to date, thus suggesting that distinct evolutionary forces underlie the conservation of PRC2 targets between plants and animals. In addition, our findings indicate that the two modes of evolutionarily conserved H3K27me3 marking that we have uncovered in the Brassicaceae are themselves subjected to distinct selective pressures.

Specifically, gene orthologs with developmentally constrained H3K27me3 marking tend to have the highest level of sequence information conservation and density of nucleosomes within their promoters. In addition, these gene orthologs have on average a higher level of H3K27me3 marking and tissue-specific expression than those with developmentally plastic marking. Thus, gene orthologs with constrained H3K27me3 marking are predominantly involved in developmental programs and include many genes encoding TFs, whereas gene orthologs with plastic H3K27me3 marking are mainly associated with metabolism and stress responses. Thus, one mode of PRC2-mediated regulation would rely on specific, hardwired promoter features that enable or facilitate tight transcriptional repression, whereas a second mode would be associated with more labile marking and less stringent gene regulation.

Given that our results were obtained from a single, complex tissue and at a defined developmental stage (fully expanded leaves of non-flowering plants [18]), it is not possible to determine if these two evolutionarily conserved modes of PRC2-mediated gene repression differ mechanistically from each other, for instance in terms of PRC2 recruitment or establishment and maintenance of the repressive state. In mammals two modes of gene regulation by PRC2 have been proposed, which differ by their mechanism of PRC2 recruitment. In the so-called instructive mode, specific TFs and long non-coding RNAs (lncRNAs) guide PRC2 to target loci, whereas in the responsive mode, PRC2 constantly samples chromatin in regulatory regions and responds to the transcriptional status of genes [3, 35, 36]. Whether similar instructive and responsive modes exist in plants and could underlie, at least in part, the developmentally constrained and plastic H3K27me3 marking modes we describe here remains to be determined. Nonetheless, it is worth mentioning that multiple PRC2 recruitment mechanisms have been proposed to operate in *Ath*, which involve notably lncRNAs and TFs [5]. Work on the TF AGAMOUS indicates that it is responsible not only for the recruitment, but also for the eviction of PRC2, depending on the target gene [37, 38], which therefore suggests a complex interplay between TFs and PRC2. In addition, the few *cis*-regulatory elements identified so far as being involved in PRC2 recruitment in *Ath* appear to be specific to individual PRC2 targets [39–41], which is consistent with our identification of GAGA and GAAGAA as the only two motifs widely shared among gene orthologs marked by H3K27me3.

We have also shown that although the two types of ancestral PRC2 targets participate in shaping 3D chromosome structure, gene orthologs with constrained H3K27me3 marking are the main contributors to strong and long-range chromosomal interactions. Thus, the involvement of H3K27me3-marked regions in intra-chromosomal interactions could represent another level of selective pressure underlying the conservation of H3K27me3 marking and the co-evolution of the genome and epigenome. Consistent with this notion, work in *Drosophila* suggests that sequence conservation collaborates with 3D chromatin architecture to maintain the evolutionary stability of Polycomb-regulated loci [13]. In other words, the regulatory information encoded in the epigenome could impose significant constraints on the evolution of genomes.

The extent to which H3K27me3 divergence results from changes in the genome sequence and vice versa is still unclear. Nonetheless, our findings suggest that tandem gene duplications could be a rapid source of new PRC2 targets. The repressive activity of PRC2 would help prevent the deleterious effects of an increase in gene dosage, thereby facilitating the subfunctionalization of gene duplicates. Finally, we have shown that, depending on their mode of H3K27me3 marking, ancestral PRC2 targets tend to be lost at different rates, thus providing further support to the notion that PRC2 activity contributes to shaping the particular evolutionary regime of target sequences.

## Conclusions

Our comparative epigenomics analysis revealed that gene orthologs marked by H3K27me3 in at least one Brassicaceae species correspond predominantly to ancestral PRC2 targets. Furthermore, we demonstrate the existence of two evolutionarily conserved modes of PRC2-mediated regulation, which are developmentally plastic and constrained, respectively. Finally, we provide evidence that the selective pressures underlying the conservation of these two modes of regulation likely operate at multiple scales, from the DNA sequence to gene function and 3D chromosome architecture.

## Methods

Biological materials, experimental and analytical procedures for ChIP-seq and RNA-seq, as well as the corresponding datasets are all described in Willing et al. (2015) [18]. Datasets are available at Gene Expression Omnibus (GEO) ([GEO:GSE50636], [GEO:GSE54727], [GEO:GSE54560]) [18].

The following datasets were also used: H3K27me3 data [8, 42–44]; Nucleosome occupancy data [22]; Hi-C data [26, 27].

These reference genome sequences were used: *Arabidopsis thaliana (Ath)* TAIR10*, Arabidopsis lyrata* (*Aly* [30]), and *Arabis alpina* (*Aal*; sequence [18] and gene annotation [18]). Numbers of annotated genes and TEs for each genome are indicated in Additional file 1: Table S11.

### Reconstruction of the Brassicaceae ancestral genome and ohnolog detection

#### Ancestral genome reconstruction

The set of *Aal* genes was included in gene families extracted from the Ensembl Genome Plants database 24 [45], and the phylogeny of each gene family was computed using the EnsemblCompara methodology [46]. Based on these gene family phylogenies, ancestral gene content was inferred for Brassicaceae. Using the Algorithm for Gene Order Reconstruction in Ancestors (AGORA) method, we automatically reconstructed contiguous ancestral regions (CARs), which describe the order (gene-to-gene adjacencies) and orientation of ancestral genes. The ancestral gene content and order we used is an update of the ancestral Brassicaceae karyotype from [16] that was computed with data from Ensembl Genomes Release 24 including four additional species: *Aal* [18] and *Tarenaya hassleriana* [19] for ingroup species and *Citrus clementina* and *Gossypium raimondii * as outgroups (both from the Phytozome 10 database [47]). All the annotations and the entire set of protein trees were downloaded from Ensembl Data Release 24. Proteins from the additional species were inserted in the previous families through an assignation based on BlastP results. Phylogenetic gene trees were computed with the EnsemblCompara methodology [46] and then used to infer the ancestral gene content, which was ordered using AGORA.

The ancestral gene content of Brassicaceae (45,874 protogenes) was extracted from the 10,840 families containing the genes present in extant Brassicaceae species. Only 9583 families contained at least one gene of *Ath*, *Aly*, or *Aal* and 6562 encompassed the set of 13,515 Brassicaceae protogenes with a single gene descendant in all three species. The reconstruction procedure led to 12 ancestral CARs (i.e., CARs with more than 400 genes) containing 19,534 ordered protogenes (out of 952 scaffolds of at least 2 genes that contain 22,885 ordered protogenes) for the Brassicaceae ancestor. This ancestral reconstruction is available in the GenomicusPlants server [48] at http://www.genomicus.biologie.ens.fr/genomicus-plants-24.01/.

All the analyses were performed on a subset of 27,343 Brassicaceae protogenes that are ancestral to *Ath*, *Aly*, and *Aal* gene orthologs, and for which the chromatin marking status is available.

#### Ohnolog detection

Ohnolog pairs of genes in the three Brassicaceae species were defined as described [49]. To identify *Aal* ohnologs, gene locations in *Aal* were compared with those of gene orthologs in non-duplicated species (*Citrus clementine*, *Theobroma cacao*, *Carica papaya*) using ad hoc scripts in order to identify pairs of regions in the *Aal* genome that are syntenic with a single region in non-Brassicaceae species. These regions correspond to double conserved synteny (DCS) blocks. Duplicate pairs of *Aal* genes were defined by their location on two distinct chromosomes belonging to the same DCS block. These genes are most likely duplicates resulting from the α- or β-WGDs and are called ohnologs. This set of ohnolog pairs is quite stringent, because only duplicates that are located on different chromosomes in extant species were taken into account, and secondly, because tandemly duplicated genes (that likely occurred after WGD) were removed from this set. The same procedure was performed for the other two species, which led to the identification of 1573, 1449, and 1780 pairs of ohnologs for *Aal*, *Ath*, and *Aly*, respectively.

### Sequence similarity estimation

For each pair of gene orthologs, conservation of the promoter (defined as the region 500 bp upstream of the transcription start site) and of the coding sequence was estimated using standard alignment-free and alignment-based methods, respectively. The alignment-free method was also used to estimate coding sequence similarity to confirm that both approaches produce the same results (Additional file 1: Table S4).

#### Alignment-based methods

We followed a conventional protocol for the estimation of sequence similarity between coding sequences. The coding sequence containing only concatenated exons, starting with a start codon (ATG) and ending with any of the three termination codons (TAA, TAG, TGA), was obtained for each gene transcript. Multiple sequence alignment of these nucleotidic sequences was computed with the TranslatorX software [31] using the corresponding amino acid translations as guides and Multiple Sequence Comparison by Log-Expectation (MUSCLE) [50] as the alignment algorithm with default parameters. The DNA distance matrix for the three sequences corresponding to each protogene was obtained with the Phylogeny Inference Package (PHYLIP) using the default F84 model [51, 52] for nucleotide substitution.

#### Alignment-free methods

Sequence similarity between non-coding sequences, like promoter sequences, cannot be appropriately computed using alignment-based methods [53, 54]. We therefore choose a *k*-mer counting method. Comparison of the frequency profiles of all *k*-mers that compose two sequences yields a small distance value when these sequences share a similar frequency profile. The following protocol was implemented as described in [55]:

- Estimation of the optimal *k*-mer length. We adopted a conservative approach and used the minimum *k*-mer length that guarantees the longest vocabulary for any sequence of length *N* composed by *c* different characters: *k*max = log_*c*_(*N*). The length of the sequences to compare varies between the promoter length (0.5 kb) and the average coding sequence length (1.2 kb); therefore, we tried both 4- and 5-mer vocabularies.
- Determination of frequency profiles. The frequency of all *k*-mers was calculated for each sequence to be compared and allocated to a vector of length 4^*k*^. High frequency *k*-mers were removed, as they tend to diminish the sensitivity of the comparisons.
- Estimation of the distance between frequency profiles using Pearson correlation and Jensen-Shannon divergence between frequency vectors.

### Correlation between chromatin marking and DNA sequence variation

We estimated the correlation between chromatin marking and DNA sequence variation using a two-way analysis of variance (ANOVA). The purpose of this analysis was to assess the extent to which chromatin marking explains the variation of DNA sequence similarity.

The response variable is the sequence similarity measured by any of the above-mentioned metrics, and the explanatory variables are:

- Concordance of marking, defined according to the number of species holding the mark for a given gene ortholog. Only orthologs with either plastic or constrained H3K27me3 marking are considered.
- Uniqueness of marking, defined as the presence of either H3K27me3 or H3K4me3 (single marking) or the co-existence of both marks (co-marking). A gene was considered as co-marked if at least one ortholog among the two species considered holds both H3K4me3 and H3K27me3 marking.

The null hypothesis is that the concordance and/or uniqueness of marking do not affect the mean sequence similarity. The analysis was done for protein coding and promoter sequence and was repeated for the two marks and the three possible pairwise comparisons: *Ath-Aly*, *Ath-Aal*, and *Aly-Aal* (Additional file 1: Table S3). Given the large number of genes tested (between O(e2) and O(e3)), ANOVA is expected to give significant results even for subtle differences in mean sequence similarity. However, even in a situation where a small variation would lead to the rejection of the null hypothesis, H3K27me3 concordance is not significantly correlated to sequence variation of the coding sequences (*p* value > 5e-2 for the alignment-based distance and *p* value > 5e-5 for the alignment-free distances). Similarly, H3K4me3 concordance is not significantly correlated to sequence variation of the promoter sequence (*p* value > 5e-5). Conversely, the omega square measure, which is an estimate of how much of the variance of the response variable is accounted for by the explanatory variables, is higher for the concordance of H3K27me3 marking with promoter sequence similarity and lower for the concordance of H3K27me3 marking with coding sequence similarity. The opposite is true for H3K4me3 marking.

### Characterization of the promoters of H3K27me3-marked gene orthologs

#### Sequence composition

Differences in the sequence composition of promoters of gene orthologs with plastic or constrained H3K27me3 marking were estimated by comparing 4-mer frequency profiles. A Z-score is calculated between the frequency of each 4-mer in promoters of plastic or constrained gene orthologs with respect to the frequency of the same 4-mer in the promoters of unmarked gene orthologs. Thus, positive and negative scores indicate 4-mers, respectively, enriched and depleted between the promoters of the different gene categories.

#### Occurrence of TEs in the promoters of H3K27me3-marked gene orthologs

The proportion of TE sequences overlapping H3K27me3-marked gene ortholog promoters was estimated for the three genomes [18]. A promoter is considered to harbor a TE sequence if the overlap between the two annotations exceeds 50% of the TE sequence length.

#### Motif finding in promoters of H3K27me3-marked gene orthologs

We searched for sequence motifs in promoters using the Multiple EM for Motif Elicitation (MEME) algorithm, which implements a probabilistic model to discover recurring ungapped fixed-length patterns from a set of unaligned sequences [56, 57]. The algorithm was run with default parameters with the following modifications: sequences may contain any number of non-overlapping occurrences of each motif; search for 5 different motifs up to 20 bp per sequence; use a Markov model of order 5 calculated over the promoters of all H3K27me3-marked gene orthologs as the background model. To assign a potential function to the predicted motifs, we compared them to a database of known Arabidopsis motifs (DNA affinity purification (DAP) motifs [58] using the Tomtom tool v4.11.2 [57]. For each motif the tool reports a set of database motifs that have significant similarity with the submitted pattern and shows the corresponding alignment. Matches were considered significant if their *q* value was smaller than 0.1.

#### Nucleosome occupancy

Average nucleosome occupancy was estimated using data obtained after micrococcal nuclease digestion of chromatin in Ath [22]. We used the nucleosome occupancy (NOC) score provided, which represents the read coverage per base of nucleosome-bound regions to calculate an average NOC score per promoter.

### Functional characterization of H3K27me3-marked gene orthologs

Functional enrichment analysis was performed using agriGO Plant GO slim [59] taking all H3K27me3-marked gene orthologs as background. Functional enrichment was estimated using the Fisher test and the Yekutieli method for multi-test *p* value adjustment; terms with an adjusted *p* value smaller than 0.05 were considered as enriched.

Tissue specificity of H3K27me3-marked gene orthologs was estimated by calculating the Shannon entropy as described [60] using publicly available transcriptome series from five different tissues (root, shoot/leaf/rosette, flower, pollen, and seed) [61]. The entropy of an expression pattern is calculated per gene using the expression level per tissue and averaging over all the tissues considered. This metric varies between 0, for genes expressed in a single tissue, and log_2_(number tissues = 5) for genes expressed uniformly in all tissues.

### H3K27me3 marking of TF-encoding gene families

TF-encoding gene families of *Ath* were retrieved from the Arabidopsis *cis*-regulatory element database (AtcisDB) of the AGRIS database. To allow meaningful comparisons, we considered only the 20 TF-encoding gene families with more than 10 members and for which at least 40% of members are among the 13,515 unambiguous gene orthologs defined initially. Additionally, promoter sequence similarity and tissue specificity of expression were estimated for genes in each TF-encoding gene family as indicated above.

### Identification and analysis of synteny blocks

Synteny blocks were determined using the PhylDiag software [29] starting from two genomes and the corresponding gene homologies. PhylDiag allows for gene deletions to be considered as events that may break the synteny and also accounts for gene orientations, tandem duplications, and lineage-specific de novo gene births. PhylDiag was run to compare the ancestral Brassicaceae genome (27,343 Brassicaceae protogenes) to each of the modern genomes (*Ath*, *Aly*, and *Aal*) with the maximum gap parameter set to 1. Using this stringent setup, we obtained 537, 497, and 867 synteny blocks for *Ath*, *Aly*, and *Aal*, which contained in total 17,442, 14,569, and 13,439 genes.

For all synteny blocks resulting from each pairwise comparison, we calculated the proportion of genes with plastic or constrained H3K27me3 marking and tested for the enrichment in each case. We performed a binomial test per synteny block using the following definitions: number of successes = number of genes with plastic or constrained H3K27me3 marking; number of trials = total number of genes in the synteny block; expected success probability = proportion of genes with plastic or constrained H3K27me3 marking averaged over all synteny blocks.

Synteny blocks enriched in gene orthologs with either plastic or constrained H3K27me3 marking were defined as those for which a significantly (*p* value < 0.05) higher proportion of successes than expected by chance was observed (Additional file 1: Table S6).

### Correlation between concordance of H3K27me3 marking, synteny, and long-distance chromosome interactions

Association between concordance of H3K27me3 marking, high intra-chromosomal connectivity, and synteny was tested as follows. We considered the co-occurrence of gene orthologs with plastic or constrained H3K27me3 marking within regions of the *Ath* genome that tend to establish more contacts than average as defined by Hi-C-seq analysis [27]. Both positive 2-kb strips with their neighboring 10-kb and topologically associating domain (TAD) interior-like regions [27] were considered as highly connected regions. We counted the number of H3K27me3-marked gene orthologs that overlap these connected regions as well as all synteny blocks that are not enriched or else enriched in gene orthologs with either plastic or constrained H3K27me3 marking. Counting was done separately for all marked genes, genes with plastic H3K27me3 marking, and genes with constrained H3K27me3 marking. Proportions were calculated by dividing these numbers by the total number of marked genes, of genes with plastic H3K27me3 marking, and of genes with constrained H3K27me3 marking, respectively.

We assessed the relation between distance and strength of genomic interactions with high density of gene orthologs with constrained H3K27me3 marking using *Ath* Hi-C-seq data obtained for the WT Col-0 and the *clf swn* mutant [26]. The distance and the strength of every intra-chromosomal interaction between 20-kb windows was inferred from the numerical matrix representation of the 2D interaction map (Fig. 1 and supplemental file S2 of Feng et al. 2014 [26] for WT and *clf swn*, respectively) and each interaction was defined as being weak, moderate, or strong. This classification was directly derived from the quantitative, color-coded, 2D interaction maps published by Feng et al. (2014) using Fig. 1 and supplemental file S2 for WT and *clf swn*, respectively [26]. Each pixel on the map represents a region of 20 kb, with *x*,*y* coordinates corresponding to physical positions along *Ath* chromosomes. The contacts are defined as follows:

- Weak contacts: regions colored from violet (0) to blue (0.59)
- Moderate contacts: regions colored from light blue (0.60) to green (1.29)
- Strong contacts: regions colored from yellow (1.30) to red (2.0) and white. Color-coded scores correspond to those depicted in Fig. 1 of Feng et al. (2014) [26].

## Additional file

Additional file 1: Contains **Figures S1**–**S9** and **Tables S1**–**S11**. (PDF 3906 kb)
