# Supplementary material for: Comparative epigenomics in the Brassicaceae reveals two evolutionarily conserved modes of PRC2-mediated gene regulation
Source: Genome Biol. 2017 Oct 31;18:207. doi: 10.1186/s13059-017-1333-9 (PMC5663038; doi:10.1186/s13059-017-1333-9)
Supplement: Additional file 1: — Contains Figures S1–S9 and Tables S1–S11. (PDF 3906 kb) [file 13059_2017_1333_MOESM1_ESM.pdf]

Figure S1

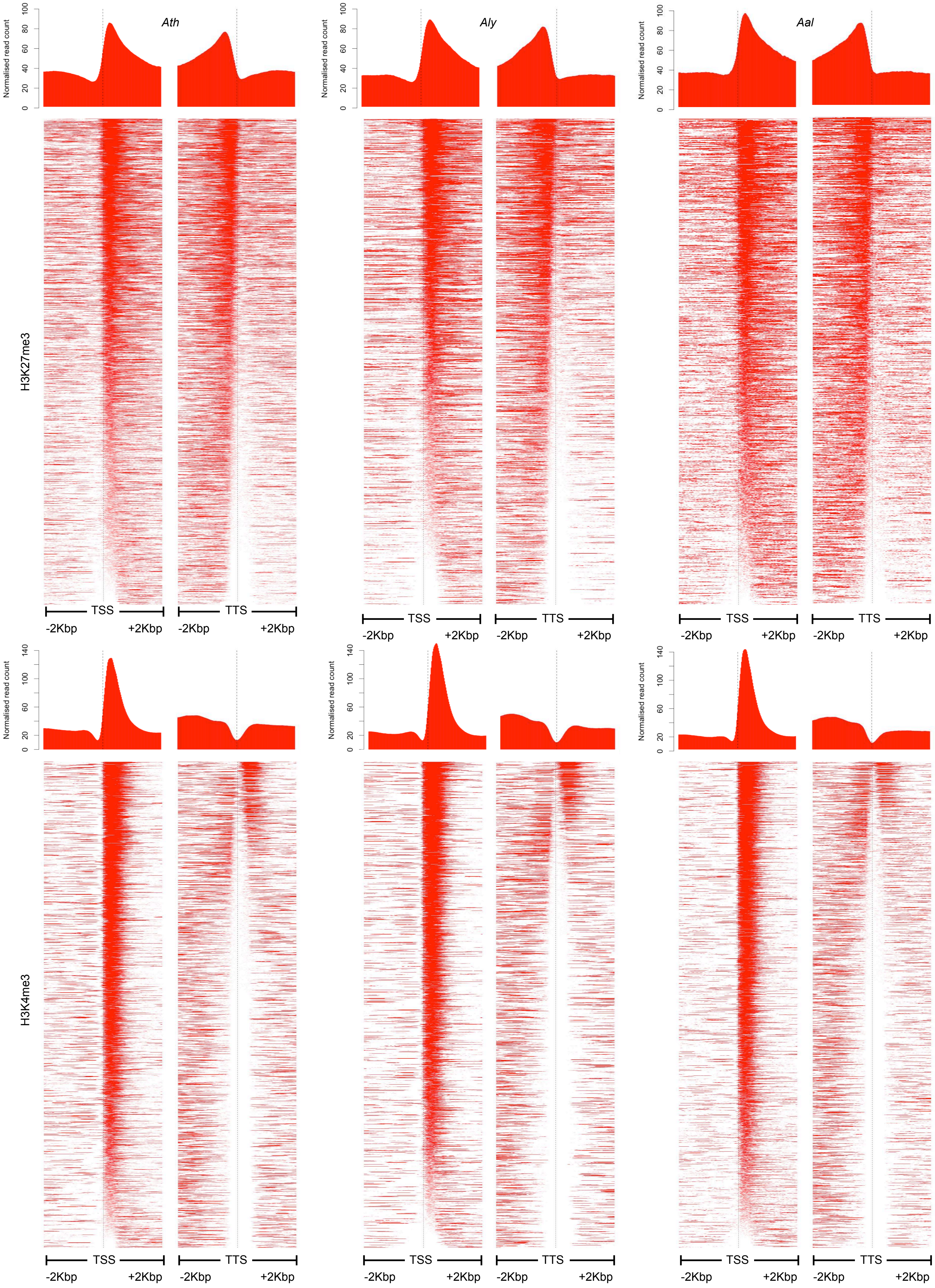

Figure S1. Pattern of H3K27me3 and H3K4me3 enrichment over marked genes.
Normalized read coverage at the transcription start (TSS) and termination sites (TTS) of all gene orthologs marked by H3K27me3 and H3K4me3 in each of the three species.

Figure S2

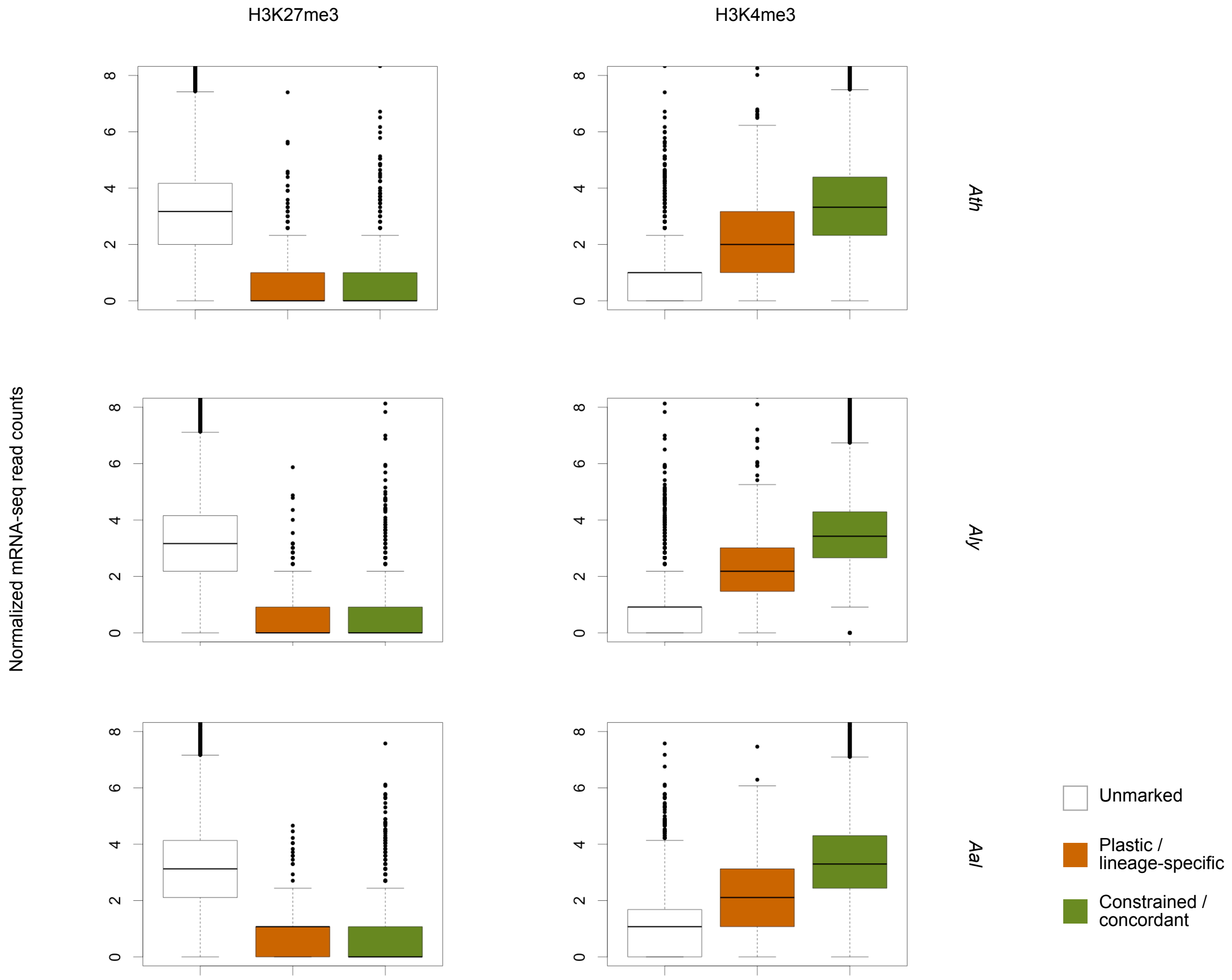

Figure S2. Average expression level of gene orthologs according to their marking status. Gene expression levels were estimated using normalized average read count per annotation. Left panels: gene orthologs with plastic or constrained H3K27me3 marking. Right panels: gene orthologs with lineage-specific or concordant H3K4me3 marking. Expression levels of unmarked genes are also shown in each panel. Gene orthologs with both H3K27me3 and H3K4me3 marking are not included in this analysis.

Figure S3

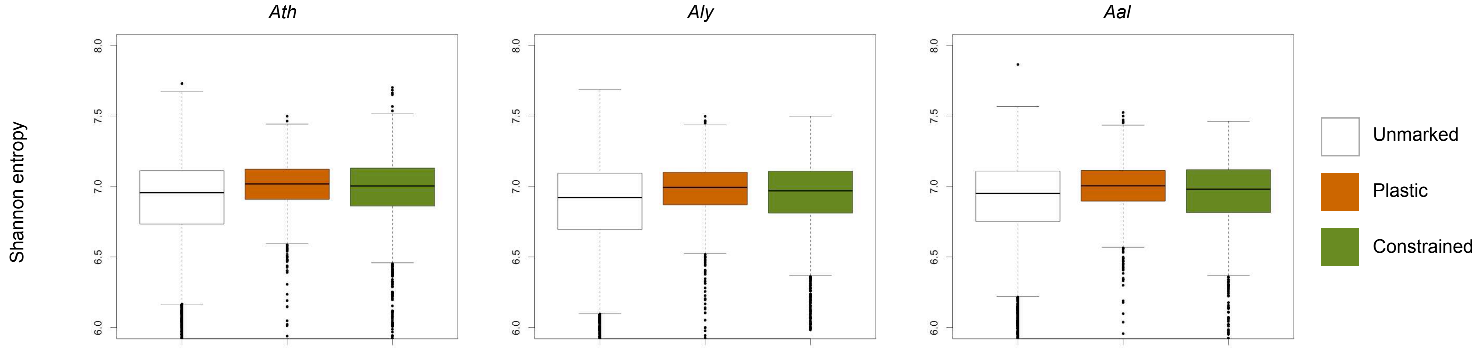

Figure S3. Shannon entropy distribution of promoter sequences of gene orthologs with plastic or constrained H3K27me3 marking.
Shannon entropy was calculated for each promoter sequence using the frequency of 4-mers and plotted for the different gene sets indicated.

Figure S4

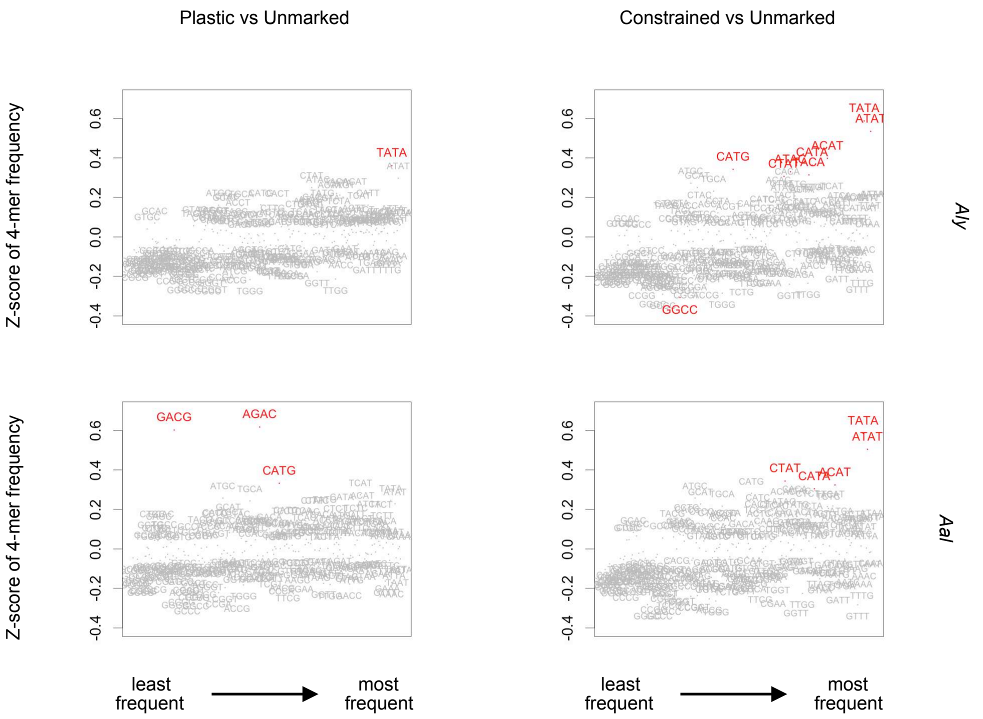

Figure S4. Sequence enrichment (4-mers) for the promoters of orthologs with plastic and constrained H3K27me3 marking in Aly and Aal.
Positive and negative Z-score values indicate higher and lower frequencies of 4-mers in comparison to unmarked gene orthologs (4-mers showing highest/lowest frequency are highlighted in red).

Figure S5

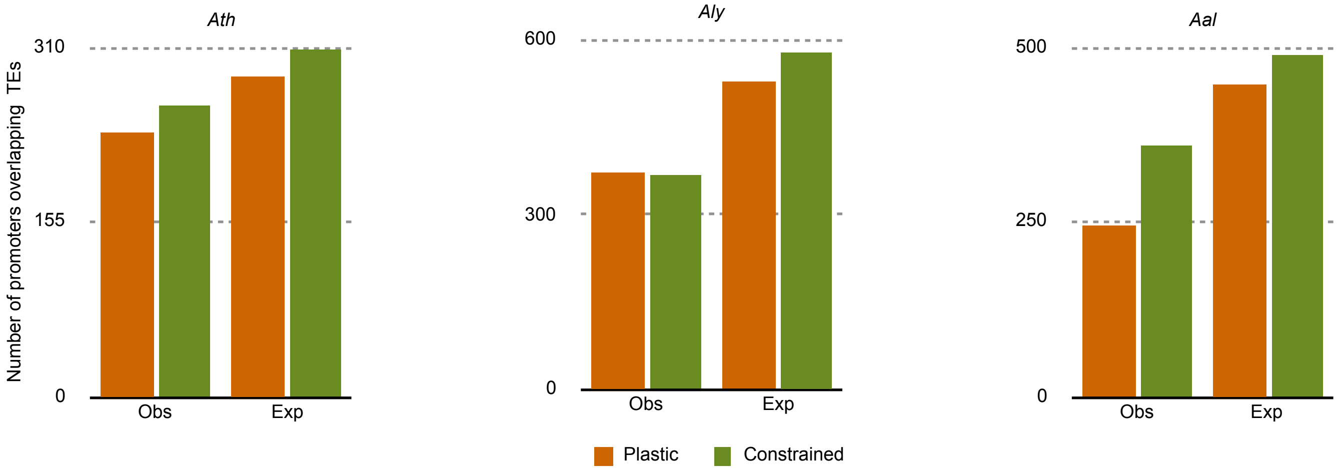

Figure S5. Transposable elements overlapping promoters of gene orthologs with plastic or constrained H3K27me3 marking.
The number of gene promoters overlapping a transposable element (TE) sequence is indicated. Both gene categories are significantly depleted in TE sequences in comparison to the expected frequency of overlap between any gene and a TE sequence genome wide (Exp; Chi-squared test, p-value<0.05).

Figure S6

A

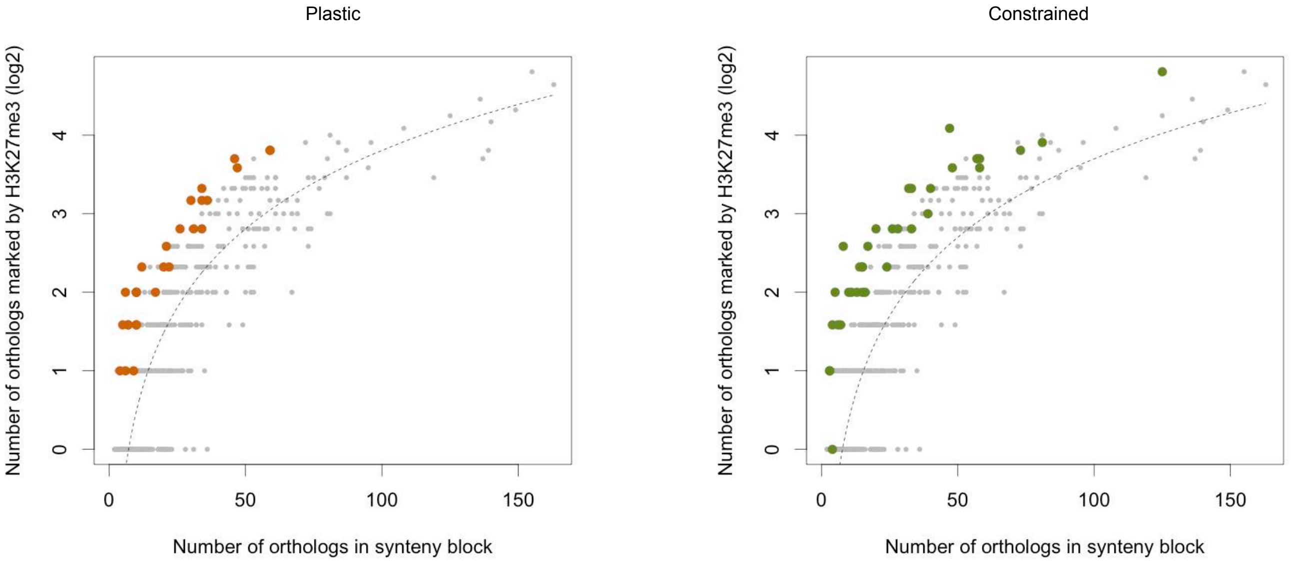

B

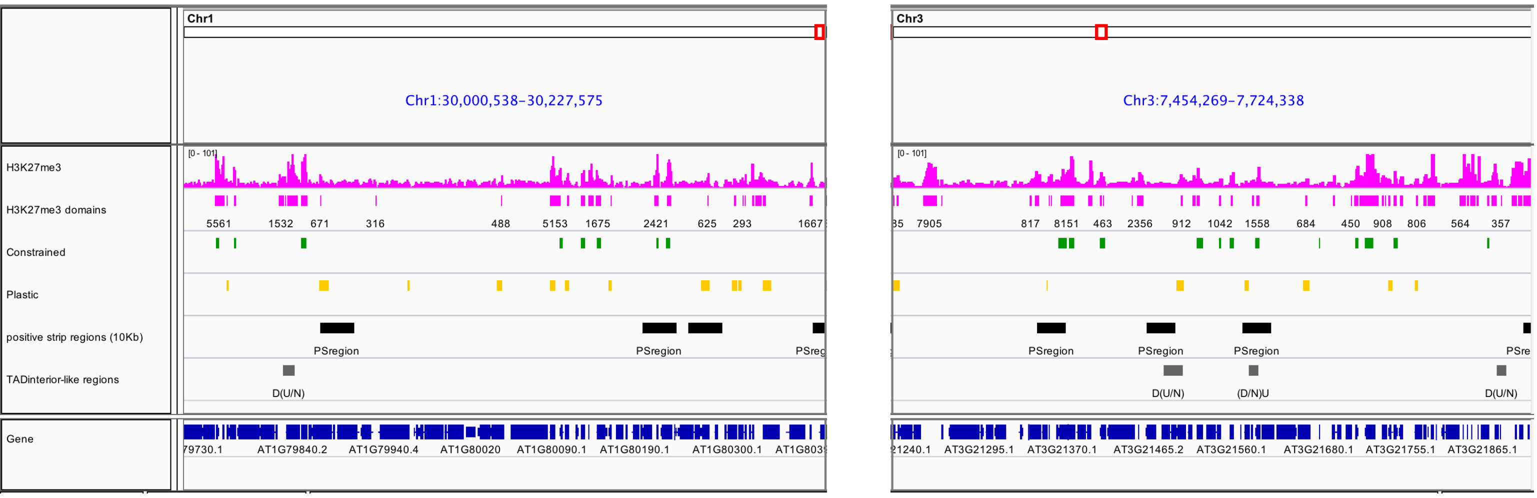

Figure S6. Enrichment of H3K27me3 marking over synteny blocks and their engagement in high connectivity regions.
A. Number of gene orthologs with either plastic or constrained H3K27me3 marking within synteny blocks. Colored dots represent synteny blocks enriched in gene orthologs with plastic (orange) or constrained (green) marking (binomial test, p-value < 0.05). B. Integrative Genomics Viewer snapshots showing a synteny block enriched in gene orthologs with plastic (left) and constrained (right) H3K27me3 marking and their overlap with the high (positive strip and TAD interior like regions) and low connectivity regions as defined in Wang et al. (2015).

Figure S7

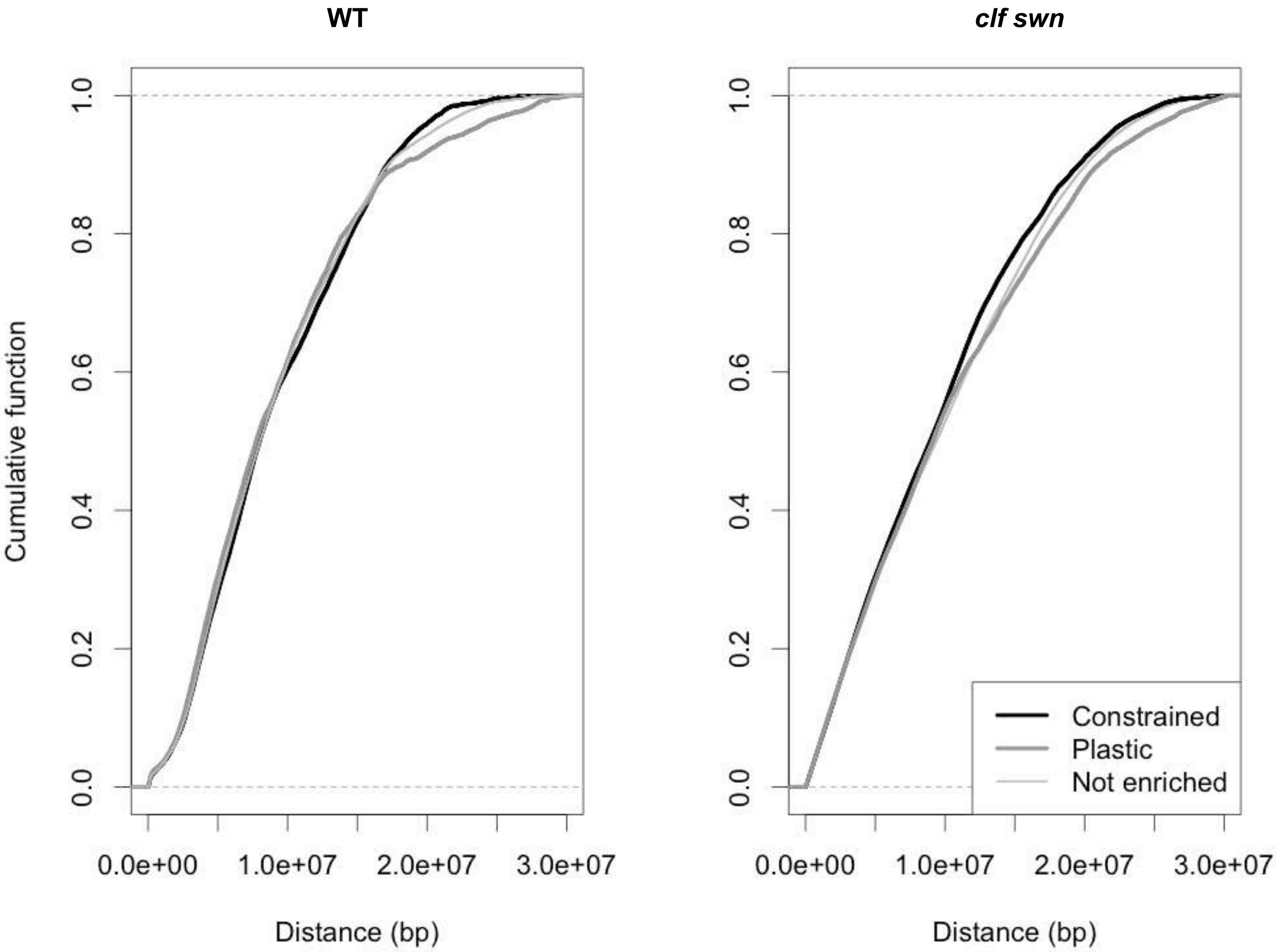

Figure S7. H3K27me3 marking of gene orthologs in relation to distance distribution of intra-chromosomal interactions in Ath.

Full-range cumulative function distribution of the length of strong intra-chromosomal contacts involving synteny blocks either not enriched or else enriched in gene orthologs with either plastic or constrained H3K27me3 marking. Results are shown for the wild type (WT) and the clf swn mutant.

Figure S8

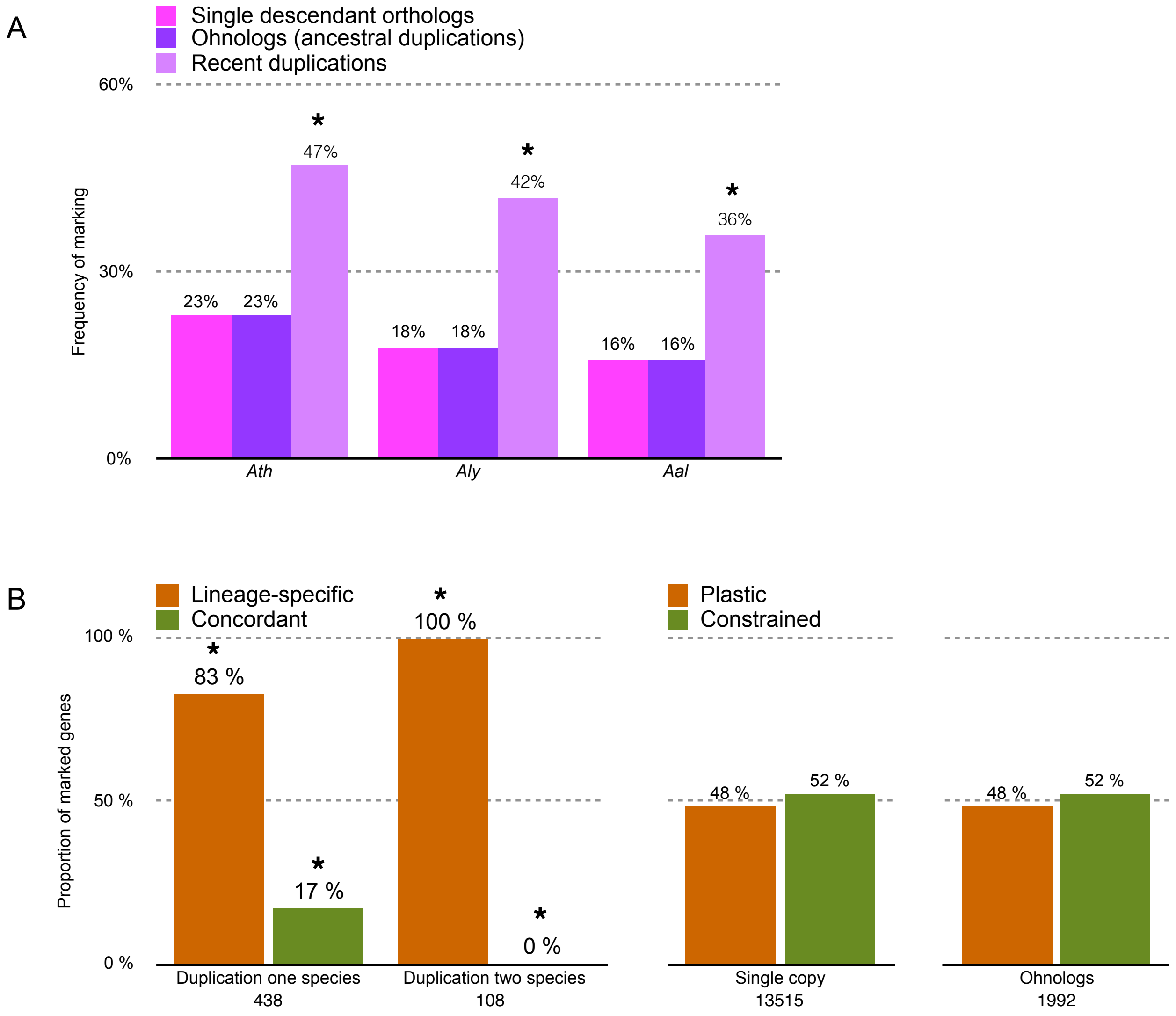

Figure S8. H3K27me3 marking patterns in relation to ancestral and recent gene duplications
A. Histograms represent the frequency of H3K27me3 marking within each species for the 13515 single descendant gene orthologs, the subset of 1992 gene ohnologs (ancient duplicates resulting fromWGDs) and the 1316 gene orthologs that have undergone recent, species-specific duplication. Asterisks indicate significant differences with the frequency of marking of single descendant gene orthologs (t-test, p-value <0.05). B. Marking concordance between species in relation to gene duplication. Left: Proportion of gene orthologs with plastic or constrained H3K27me3 marking for single descendant gene orthologs and the subset of ohnologs. Right: Proportion of gene orthologs with lineage-specific or concordant marking for genes that have undergone recent species-specific duplication. The total number of genes included in the quantification is indicated below each graph. Asterisks indicate significant differences with the frequency of marking for single descendant gene orthologs (t-test, p-value <0.05). For the quantification over the complete set of 27343 Brassicaceae protogenes, see Additional file 1: Table S10.

Figure S9

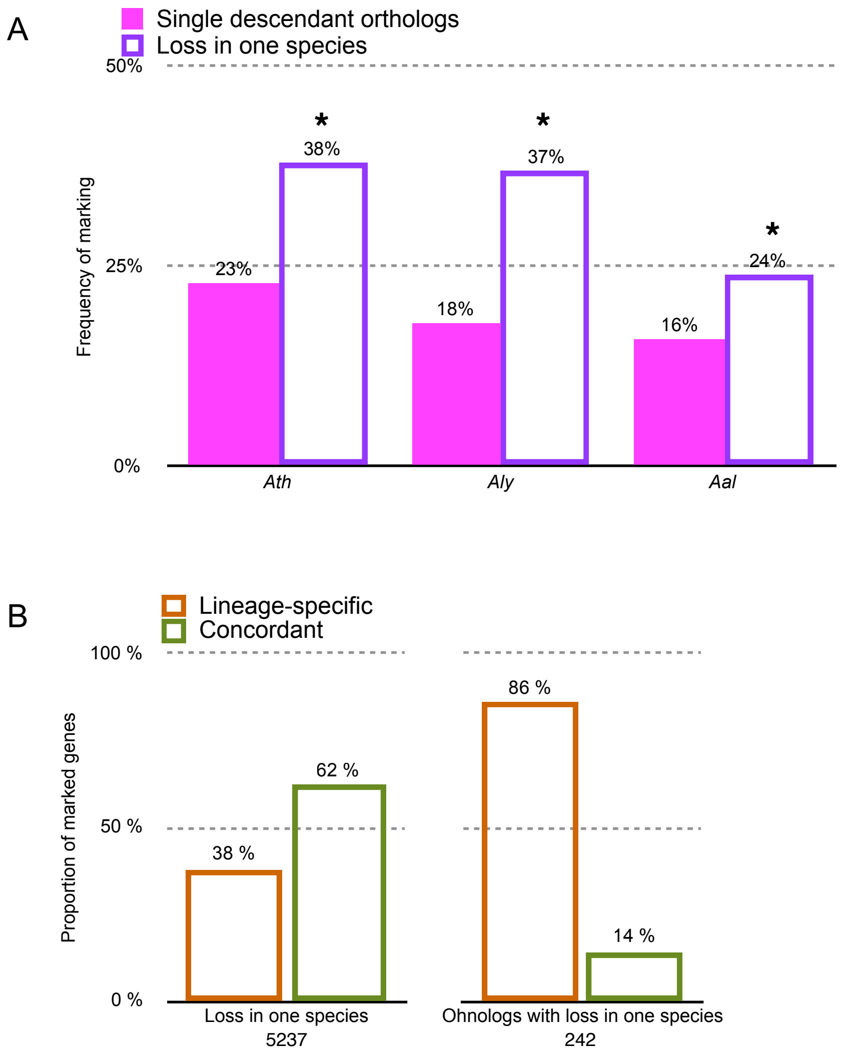

Figure S9. H3K27me3 marking patterns in relation to gene loss.
A. Histograms represent the frequency of H3K27me3 marking within each species for the set of 13515 single descendant gene orthologs present in all three species and the set of 12512 gene orthologs that have no representative (gene loss) in one species. Asterisks indicate significant differences with the frequency of marking of single descendant gene orthologs (t-test, p-value <0.05). B. Marking concordance between species in relation to gene loss. Proportion of gene orthologs with lineage-specific or concordant H3K27me3 marking for gene orthologs and the 242 for which ohnologs can be identified. The total number of gene orthologs included in the quantification is indicated below each graph. For the quantification over the complete set of 27343 Brassicaceae protogenes, see Additional file 1: Table S10.

Table S1. Number of genes marked by H3K4me3 and H3K27me3 per species

Marking	*Ath*	*Aly*	*Aal*
**H3K27me3 only**	5720	5196	4563
**H3K4me3 only**	15116	15812	16921
**H3K27me3 and H3K4me3**	2726	2033	1970
**Unmarked**	4934	4541	3711

Table S2. Observed and expected marking frequencies among species

H3K27me3 marking	Expected	Observed
**3 species**	1113 (30%)	1464 (40%)
**2 species**	1737 (48%)	806 (22%)
**1 species**	823 (22%)	1403 (38%)

Chi-squared test, p-value< 10^{-5}

Table S3. Distribution of gene orthologs with constrained or plastic H3K27me3 marking among gene families

H3K27me3-marked genes present in families	Number of gene families	Total number of PRC2 targets	
		Constrained	Plastic
**Only genes with constrained marking**	688	1077	0
**Only genes with plastic marking**	1119	0	1330
**More genes with constrained than plastic marking**	118	357	144
**More genes with plastic than constrained marking**	86	105	222
**Equal number of genes with constrained and plastic marking**	184	219	219
**3673 genes present in the set of the 13515 genes corresponding to single gene descendants in all three species**	2195	1758	1915

Table S4. ANOVA table for factors affecting coding and promoter sequence conservation per mark and species.

Sequence	Distance	Comparison	Mark	p-value			Omega squared	
				Con	Unq	Con:Unq	Con	Unq
Coding	DNA dist	*Ath-Aly*	H3K27me3	5.0E-01	9.9E-02	4.7E-01	-0.000	0.001
	DNA dist	*Ath-Aal*	H3K27me3	4.9E-01	6.2E-03	3.4E-01	-0.000	0.003
	DNA dist	*Aly-Aal*	H3K27me3	6.2E-01	8.4E-02	8.4E-01	-0.000	0.001
	DNA dist	*Ath-Aly*	H3K4me3	9.9E-03	1.1E-03	5.2E-01	0.001	0.001
	DNA dist	*Ath-Aal*	H3K4me3	1.1E-09	5.6E-22	2.1E-01	0.003	0.009
	DNA dist	*Aly-Aal*	H3K4me3	9.3E-10	2.3E-29	1.5E-02	0.003	0.012
	4-mer JSD	*Ath-Aly*	H3K27me3	1.6E-01	5.0E-09	1.2E-05	0.000	0.010
	4-mer JSD	*Ath-Aal*	H3K27me3	3.8E-02	1.1E-04	3.4E-03	0.001	0.005
	4-mer JSD	*Aly-Aal*	H3K27me3	9.6E-01	3.7E-10	6.6E-01	-0.000	0.012
	4-mer JSD	*Ath-Aly*	H3K4me3	4.5E-21	1.4E-17	1.7E-01	0.007	0.007
	4-mer JSD	*Ath-Aal*	H3K4me3	6.2E-28	3.4E-32	5.1E-02	0.010	0.012
	4-mer JSD	*Aly-Aal*	H3K4me3	3.1E-17	2.8E-38	1.5E-03	0.006	0.014
	4-mer PC	*Ath-Aly*	H3K27me3	6.3E-02	9.0E-08	2.6E-05	0.001	0.009
	4-mer PC	*Ath-Aal*	H3K27me3	2.4E-03	1.2E-04	7.0E-03	0.002	0.005
	4-mer PC	*Aly-Aal*	H3K27me3	7.7E-01	1.0E-11	6.6E-01	-0.000	0.014
	4-mer PC	*Ath-Aly*	H3K4me3	6.3E-17	6.5E-19	9.0E-01	0.006	0.007
	4-mer PC	*Ath-Aal*	H3K4me3	3.3E-25	4.4E-34	1.6E-01	0.009	0.013
	4-mer PC	*Aly-Aal*	H3K4me3	2.3E-16	3.3E-44	1.2E-02	0.005	0.017
Promoter	4-mer JSD	*Ath-Aly*	H3K27me3	2.9E-19	5.8E-01	5.6E-01	0.022	-0.000
	4-mer JSD	*Ath-Aal*	H3K27me3	8.3E-15	1.6E-01	1.8E-04	0.016	0.001
	4-mer JSD	*Aly-Aal*	H3K27me3	7.1E-20	6.2E-04	2.5E-01	0.022	0.004
	4-mer JSD	*Ath-Aly*	H3K4me3	2.2E-01	1.3E-09	4.4E-01	0.000	0.004
	4-mer JSD	*Ath-Aal*	H3K4me3	5.4E-01	4.5E-19	2.2E-01	-0.000	0.007
	4-mer JSD	*Aly-Aal*	H3K4me3	1.6E-02	3.6E-17	3.7E-02	0.000	0.007
	4-mer PC	*Ath-Aly*	H3K27me3	2.3E-25	6.0E-01	3.7E-01	0.031	-0.000
	4-mer PC	*Ath-Aal*	H3K27me3	2.9E-21	6.6E-01	8.7E-03	0.025	-0.000
	4-mer PC	*Aly-Aal*	H3K27me3	2.1E-25	3.7E-02	7.7E-01	0.031	0.002
	4-mer PC	*Ath-Aly*	H3K4me3	1.1E-01	1.4E-25	8.6E-02	0.000	0.012
	4-mer PC	*Ath-Aal*	H3K4me3	7.6E-01	6.9E-32	4.8E-01	-0.000	0.015
	4-mer PC	*Aly-Aal*	H3K4me3	2.9E-02	4.8E-32	2.3E-03	0.000	0.015

4-mer = k-mers of length 4
PC = Pearson correlation
JSD = Jensen-Shannon divergence
Con = Concordance of marking
Unq = Uniqueness of marking
Con:Und = interaction between concordance and uniqueness

Table S5. Motif analysis of promoter sequences in each H3K27me3-marking category per species

Species	H3K27me3 marking	Motif	E-value	Known motifs DAP DB
Ath	Constrained	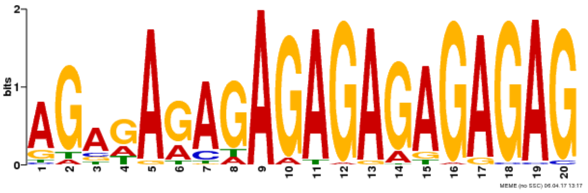	6.0e-184	
		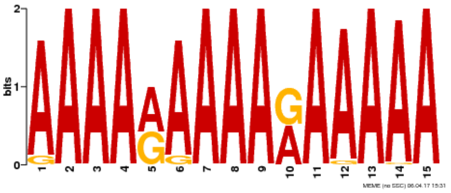	1.3e-055	
		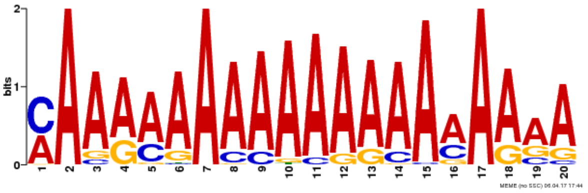	1.3e-052	
		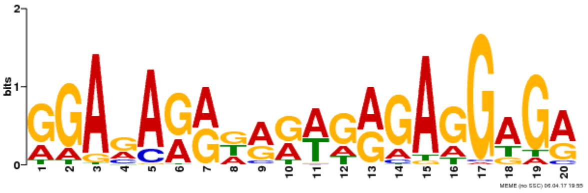	2.4e-026	
		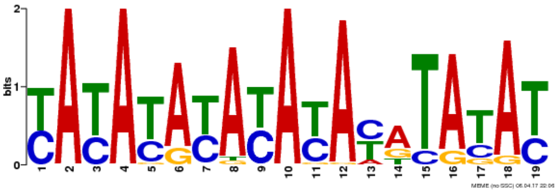	6.4e-028	
Ath	Plastic	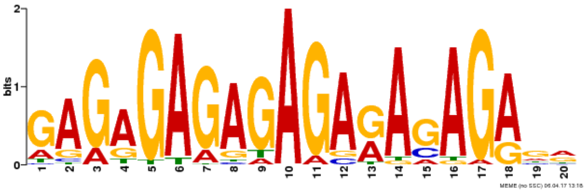	5.1e-102	
		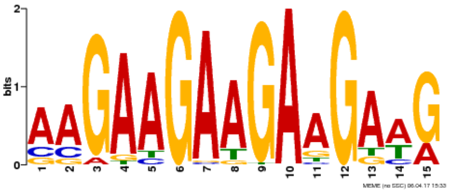	4.2e-039	
		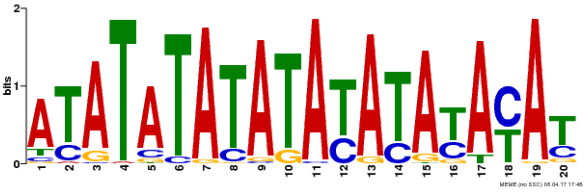	5.2e-044	

		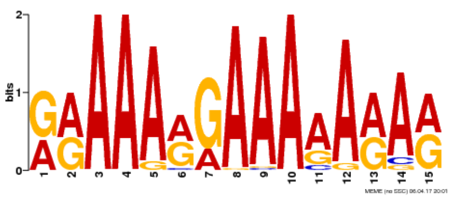	3.1e-015	
Aly	Constrained	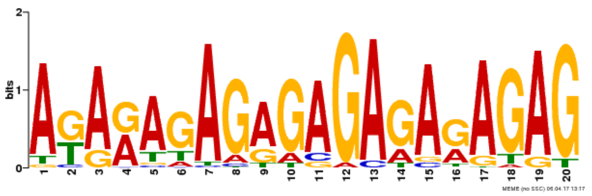	2.4e-094	
		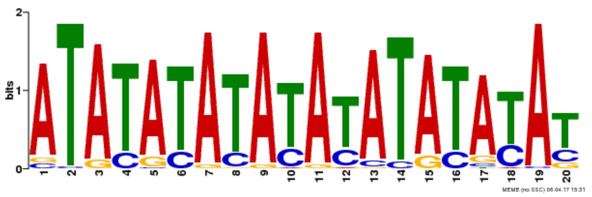	1.0e-054	
		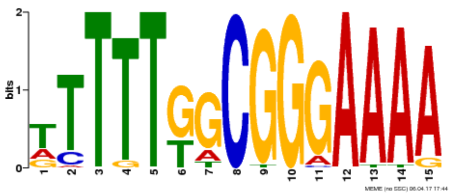	8.6e-051	E2F-DP, ABI3VP1, ARF, AP2EREBP
		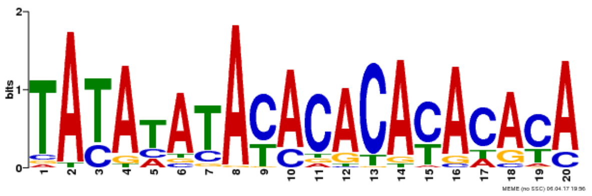	2.9e-028	
		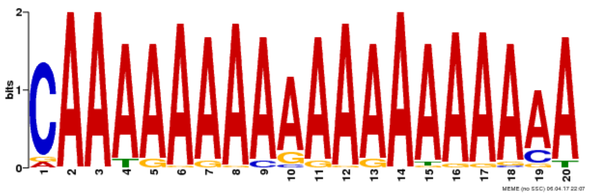	4.9e-119	
Aly	Plastic	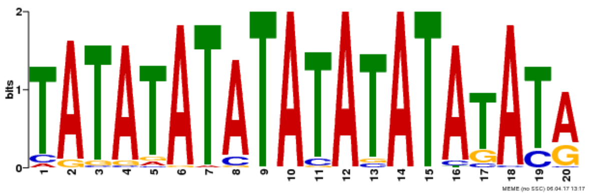	9.3e-076	
		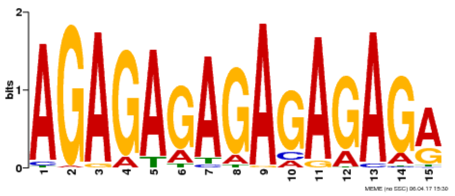	4.1e-074	

		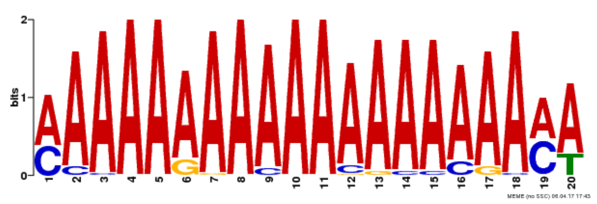	2.4e-111	
		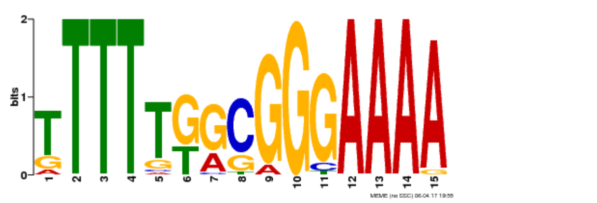	1.9e-019	E2F-DP, ABI3VP1, ARF, AP2EREBP
		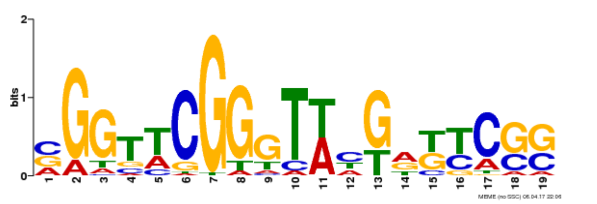	7.4e-006	MYB
Aal	Constrained	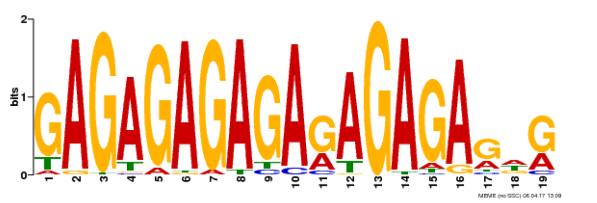	6.9e-145	
		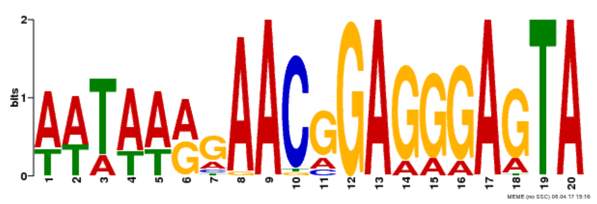	4.0e-059	
		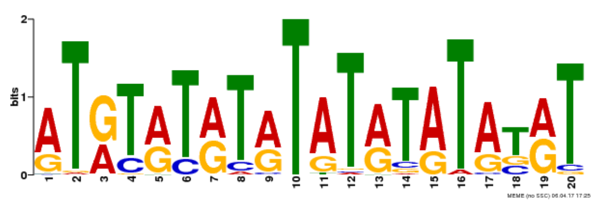	7.1e-029	
		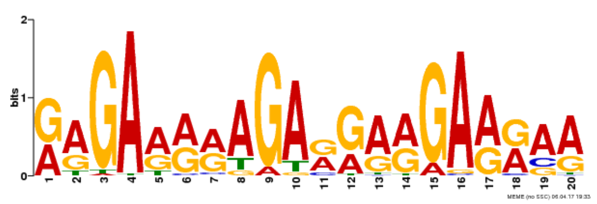	4.8e-044	
		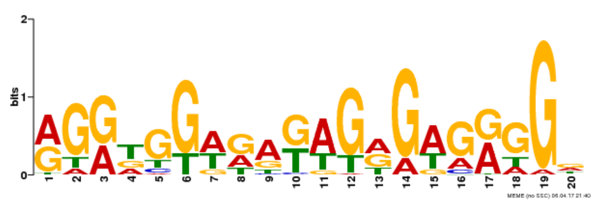	3.6e-007	

Aal	Plastic	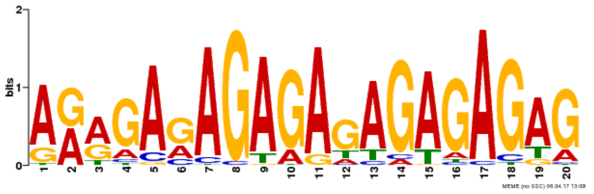	2.1e-098	
		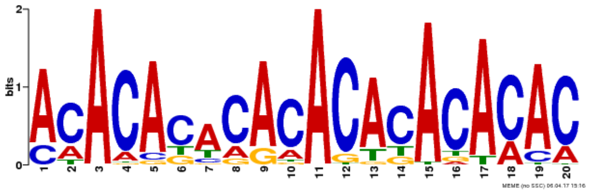	3.5e-079	
		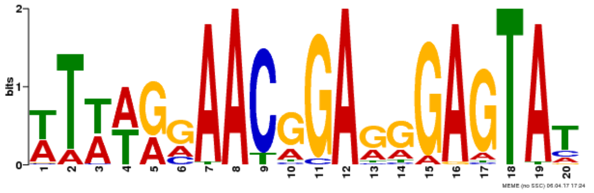	1.6e-061	
		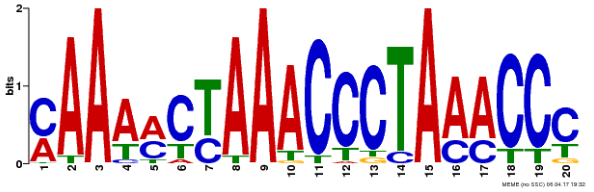	4.3e-045	MYB related, SRS
		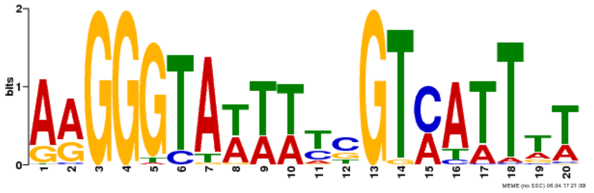	3.0e-049	

Table S6. Number of genes orthologs in the three genomes and in synteny blocks

Gene type	Whole genome	Synteny blocks		
		Ath	***Aly***	***Aal***
**Protogenes**	27343	13522	16218	13522
**With H3K27me3 marking**	5229, 4484, 3545*	4506	3372	2280
**Constrained H3K27me3 marking**	1758	1567	1561	1293
**Plastic H3K27me3 marking**	1915	1688	1685	1436
**Unmarked**	9842	9075	8998	7724

* Total number of protogenes with H3K27me3 marking per genome: *Ath, Aly, Aal*

Table S7. Number of synteny blocks with differential enrichment in gene orthologs with H3K27me3 marking

Synteny block enrichment	*Ath*	*Aly*	*Aal*
**Constrained H3K27me3 marking**	34	32	63
**Plastic H3K27me3 marking**	28	25	39
**Not enriched**	475	440	765

Table S8. Co-occurrence of high-connectivity regions with genes marked by H3K27me3 in *Ath* synteny blocks

H3K27me3 marking	Total number of genes			Number of genes overlapping High-connectivity regions		
	Synteny block enrichment			Synteny block enrichment		
	Constrained	Plastic	Not enriched	Constrained	Plastic	Not enriched
**Constrained**	238	62	1267	108	19	469
**Plastic**	126	155	1407	42	56	359
**Unmarked**	578	365	8132	127	52	1479

Table S9. Contact distribution of *Ath* synteny blocks

Synteny block enrichment	Total size (bp)	Number of contacts between 20 Kb windows - WT			Number of contacts between 20 Kb windows - *clf swn*		
		Strong	Medium	Weak	Strong	Medium	Weak
**Constrained H3K27me3 marking**	7882021	91460	347469	78431	257518	0	259842
**Plastic H3K27me3 marking**	4048074	48437	190197	40958	138756	0	140836
**Not enriched**	82150332	1028100	3600419	883999	2746930	0	2765588
**Total**	94080427	1167997	4138085	1003388	3143204	0	3166266

Table S10. Marking concordance for all gene orthologs

H3K27me3 marking concordance	Gene orthologs with no recent duplicates							Gene orthologs with recent duplicates					
	Single copy	With loss		Ohnologs				Dup in three sp	Dup in two sp	Dup in one sp	Dup in two sp	Dup in one sp	Dup in one sp
	No loss	Loss in one sp	Loss in two sp	No loss	Loss in one sp	Loss in two sp	Loss in one/two sp	No loss			Loss in one sp		Loss in two sp
Constrained / concordant *	1758	3051	-	268	9	-	103	9	15	0	2	0	-
Plastic / lineage specific *	1915	1366	4976	288	57	31	308	5	71	214	96	242	159
Unmarked	9842	820	2299	1436	176	137	1077	11	22	224	21	111	114
Total	13515	5237	7275	1992	242	168	1488	25	108	438	119	353	273
Total by ancestral gene set	13515	12512		3890				1316					

* Constrained and plastic H3K27me3 marking categories were defined only for the set of 13515 single-descendant gene orthologs. For orthologs with recent duplication or loss in one or two species the marking concordance is defined as lineage-specific (marked only in one species or in *Arabidopsis sp.*) or concordant (marked in three species for gene orthologs with recent duplicates or marked in two species for gene with loss in one species).
sp = species

Table S11. Reference genomes and annotated features

**Annotation**	***Ath***	***Aly***	***Aal***
**Chromosomes / super-scaffolds**	5 (119Mb)	8 (195Mb)	8 (235Mb)
**Genes**	28496	27582	27165
**Transposable elements**	23322	47396	71937
